# Management of hypertension and renin-angiotensin-aldosterone system blockade in adults with diabetic kidney disease: Association of British Clinical Diabetologists and the Renal Association UK guideline update 2021

**DOI:** 10.1186/s12882-021-02587-5

**Published:** 2022-01-03

**Authors:** D. Banerjee, P. Winocour, T. A. Chowdhury, P. De, M. Wahba, R. Montero, D. Fogarty, A. H. Frankel, J. Karalliedde, P. B. Mark, D. C. Patel, A. Pokrajac, A. Sharif, S. Zac-Varghese, S. Bain, I. Dasgupta, On behalf of the Association of British Clinical Diabetologists and The Renal Association

**Affiliations:** 1 St George’s Hospitals NHS Foundation Trust, London, UK; 2 ENHIDE, East and North Herts NHS Trust, Stevenage, UK; 3 Royal London Hospital, London, UK; 4 City Hospital, Birmingham, UK; 5 St Helier Hospital, Carshalton, UK; 6 King’s College, London, UK; 7 Belfast Health and Social Care Trust, Belfast, UK; 8 Imperial College Healthcare NHS Trust, London, UK; 9 Guy’s and St Thomas’ Hospital London, London, UK; 10 University of Glasgow, Glasgow, UK; 11 Royal Free London NHS Foundation Trust, London, UK; 12 West Hertfordshire Hospitals, London, UK; 13 University Hospitals Birmingham NHS Foundation Trust, Birmingham, UK; 14 Swansea University, Swansea, UK

**Keywords:** Diabetes, Hypertension, Chronic kidney disease, dialysis, ACE inhibitors, Angiotensin receptor blockers

## Abstract

People with type 1 and type 2 diabetes are at risk of developing progressive chronic kidney disease (CKD) and end-stage kidney failure. Hypertension is a major, reversible risk factor in people with diabetes for development of albuminuria, impaired kidney function, end-stage kidney disease and cardiovascular disease. Blood pressure control has been shown to be beneficial in people with diabetes in slowing progression of kidney disease and reducing cardiovascular events. However, randomised controlled trial evidence differs in type 1 and type 2 diabetes and different stages of CKD in terms of target blood pressure. Activation of the renin-angiotensin-aldosterone system (RAAS) is an important mechanism for the development and progression of CKD and cardiovascular disease. Randomised trials demonstrate that RAAS blockade is effective in preventing/ slowing progression of CKD and reducing cardiovascular events in people with type 1 and type 2 diabetes, albeit differently according to the stage of CKD. Emerging therapy with sodium glucose cotransporter-2 (SGLT-2) inhibitors, non-steroidal selective mineralocorticoid antagonists and endothelin-A receptor antagonists have been shown in randomised trials to lower blood pressure and further reduce the risk of progression of CKD and cardiovascular disease in people with type 2 diabetes. This guideline reviews the current evidence and makes recommendations about blood pressure control and the use of RAAS-blocking agents in different stages of CKD in people with both type 1 and type 2 diabetes.

**Supplementary Information:**

The online version contains supplementary material available at 10.1186/s12882-021-02587-5.

## Introduction

A significant percentage of people with diabetes develop chronic kidney disease (CKD), and diabetes is also a leading cause of end-stage kidney disease [1]. Nearly a third of people who are on dialysis in the UK have diabetes [2]. Diabetic kidney disease (DKD), an umbrella term used to describe diabetic nephropathy and CKD in diabetes, is associated with high morbidity and mortality, predominantly related to cardiovascular complications, and the progression to kidney failure requiring renal replacement therapy. Hypertension is a modifiable risk factor for cardiovascular complications and progression of CKD [3].

Angiotensin converting enzyme inhibitors (ACEi) and angiotensin receptor II blockers (ARB) are established treatment to slow the progression of DKD and reduce cardiovascular events. Novel agents such as sodium glucose cotransporter-2 (SGLT-2) inhibitors, non-steroidal selective mineralocorticoid receptor antagonists and endothelin A receptor antagonists have recently been demonstrated to improve clinical outcomes and lower blood pressure, and are likely to be used in the routine management of DKD in the future [4].

The scope of this update includes lifestyle advice, blood pressure targets and antihypertensive therapies in different categories of patients with DKD. Accurate measurement of blood pressure is vitally important before starting and during monitoring of antihypertensive treatment. Separate recommendation for blood pressure measurement has not been made in this guidance. We suggest the British and Irish Hypertension Society’s (BIHS) guidance on standardised, automated blood pressure measurement is followed. The blood pressure thresholds and targets in this guideline refer to standardised office blood pressure readings unless specified otherwise.

This guidance is for a variety of clinicians who treat people with diabetic kidney disease, including primary care physicians and specialists in diabetes, cardiology and nephrology. It intends to harmonise practices of blood pressure monitoring, and pharmacological and non-pharmacological management of hypertension, which vary considerably in different settings.

**Fig. 1:**
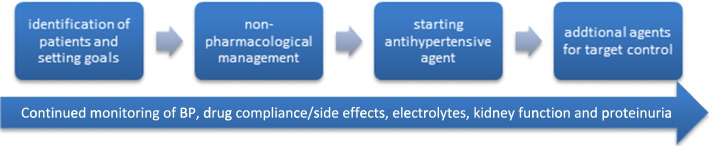
Steps in the management of hypertension in people with diabetes and CKD

The guideline provides separate recommendations for type 1 and type 2 diabetes, with type 2 further divided into early (CKD stages 1–3) as well as advanced CKD (CKD stages 4–5), and dialysis patients (see Table 1, Fig. 1). The diagnosis and management of post solid organ transplantation diabetes have been discussed in a separate guidance. The main research recommendations appear as a separate section and the audit standards are included in the supplementary file.

**Table 1:** Blood pressure targets in people with diabetes through stages of kidney function impairment

	Stage of kidney function impairment				
	Normal kidney function, normoalbuminuria	Normal kidney function, microalbuminuria	CKD stages 1–3	CKD stages 4–5 (non-dialysis)	CKD stage 5 (dialysis)
**Type 1 diabetes in mmHg (evidence grade)**	< 140/80–90 (2D) < 120/80 (2D)^d^ (for< 30 years)	≤130/80 (1B) 120/80 (2D)^d^	≤130/80 (1B) 120/80 (2D)^d^	≤140/90 (1B) ≤130/80 for albuminuric(2C)	≤140/90 (2D)^c^ (interdialytic BP)
**Type 2 diabetes in mmHg (evidence grade)**	< 140/90 (1D) < 150/90 (2B)^b^ (for ≥75 years)	< 130/80 (2D)	< 130/80 (2D)	< 140/90 (1B)^a^ < 130/80 for albuminuric (2C)	< 140/90 (2D)^c^ (interdialytic BP)

*CKD* chronic kidney disease, *BP* blood pressure

^a^For adults > 65 years a higher target > 140/90 may be appropriate

^b^For frail adults > 75 years a higher target > 150/90 may be appropriate to avoid side effects

^c^Monitor and target inter-dialytic home BP for people on dialysis

^d^ Lower targets for younger adults aged < 30

### Hypertension management and renin-angiotensin-aldosterone system blockade in people with type 1 diabetes with CKD stages 1–5 non dialysis

#### Recommendations (Table 2)

Despite improvements in prognosis, diabetic nephropathy in people with type 1 diabetes remains a major cause of end-stage renal disease [5]. The onset of micro- and macroalbuminuria in people with type 1 diabetes heralds not only an increased risk of renal disease, but also an increased risk of cardiovascular disease [6]. Early prospective studies suggested that around 30–50% of people with type 1 diabetes will develop microalbuminuria, in whom a 6% increase in risk of coronary heart disease is seen per 5 mg increase in 24-h albumin excretion rate (AER) [6]. The natural history of diabetic nephropathy in people with type 1 diabetes has, however, changed over the past 4 decades. Studies in the 1970s and 1980s suggested that progression to end-stage kidney disease in people with macroalbuminuria would occur within 7 years [7]. More recent follow-up data of significant numbers of people with type 1 diabetes suggest that end-stage kidney disease occurs in around 3% of people who have had diabetes for 10 years [8] and in around 8% of people who have had diabetes for 30 years [9].

**Table 2:** Recommendations for people with type 1 diabetes

Recommendations for renin-angiotensin-aldosterone system (RAAS) blockade and hypertension management in people with type 1 diabetes	
1. a. In people with type 1 diabetes and urine albumin:creatinine ratio (UACR) < 3 mg/mmol [< 26.55 mg/g]), we recommend a threshold for blood pressure therapy of a persistent upright (sitting or standing) blood pressure that is ≥140/90 mmHg (1B)^a, b^. b. In children and adolescents with type 1 diabetes, hypertension is defined as average systolic blood pressure and/or diastolic blood pressure that is greater than the 95th percentile for the person’s gender, age and height on more than three occasions (Grade 1B). 2. We recommend that angiotensin-converting-enzyme inhibitor (ACEI) therapy should be used as a first-line agent for blood pressure lowering and, if ACEI therapy is contraindicated or not tolerated, angiotensin receptor blockers (ARBs) should be considered (Grade 1B). 3. In most adults with type 1 diabetes and persistent UACR > 3 mg/mmol (> 26.55 mg/g), we recommend that ACEI therapy should be considered irrespective of blood pressure, and that the target upright blood pressure should be ≤130/80 mmHg (1B) if higher pre-treatment in younger adults but ≤140/90 mmHg for those aged over 65 (2D). We recommend that the dose of ACEI should be titrated to the maximum tolerated (Grade 1B). 4. There is no current evidence to support a role for ACEI therapy for blood pressure control or renal protection in people with type 1 diabetes who are normotensive and have UACR < 3 mg/mmol [< 26.55 mg/g]) (Grade 1C). 5. There is some evidence to support the use of candesartan to prevent the development or progression of retinopathy in people with type 1 diabetes who are normotensive and have UACR < 3 mg/mmol [< 26.55 mg/g]) (Grade 1C). 6. There is no firm evidence to support a role of dual blockade of the RAAS in people with type 1 diabetes (Grade 1C). 7. We recommend that people with type 1 diabetes should be advised to stop RAAS-blocking drugs during periods of acute illness and restart on recovery (Grade 1C). 8. We recommend that women of childbearing age should be encouraged to stop RAAS-blocking drugs prior to actively considering pregnancy (Grade 1B).	

^a^We suggest a target upright blood pressure in younger adults of 120/80 mmHg and 140/90 mmHg for those aged over 65 (Grade 2D)

^b^Between the ages of 30–65 for some people with higher life-time risk through earlier age of onset of type 1 diabetes, it may be appropriate to target a diastolic BP of < 80 mmHg (Grade 2C)

There is a significant body of evidence to suggest that over-activation of the RAAS plays a major role in the pathogenesis of diabetic nephropathy in people with type 1 diabetes [10]. Over-activation of the RAAS is observed in people with type 1 diabetes, even in the absence of diabetic nephropathy [11]. Angiotensin II-mediated increase in intraglomerular pressure appears to be an important mechanism by which renal disease progresses in people with type 1 diabetes who have diabetic nephropathy [12, 13], and reductions in intraglomerular pressure may ameliorate glomerular injury. Angiotensin II also has mitogenic effects that may lead to mesangial expansion that is characteristic of diabetic nephropathy [14]. Over-activation of the RAAS may be mediated by hyperglycaemia [15], and blockade of the RAAS may in turn be impaired by hyperglycaemia [13]. RAAS over-activation is also described in people with type 1 diabetes who have glomerular hyperfiltration [16].

#### Hypertension in people with type 1 diabetes

Risk factors for the development of nephropathy in people with type 1 diabetes include increasing age, duration of diabetes, male gender and hyperglycaemia [17]. The possible role of genetic factors has long been hypothesised, due to the observation that a family history of hypertension appears to predict the development of nephropathy [18]. It has been suggested that a family history of hypertension could be the basis for more intensive antihypertensive therapy in people with type 1 diabetes.

The risk factor that has the strongest association with progression of diabetic nephropathy is hypertension. Prospective evaluation of 148 people with type 1 diabetes who were normoalbuminuric showed that those who developed microalbuminuria had a significantly higher baseline blood pressure compared with those who remained normoalbuminuric (138/82 mmHg versus 123/73 mmHg) [19]. Similarly, follow-up of a Scandinavian cohort of over 300 children and adolescents with type 1 diabetes showed that systolic blood pressure was a major risk factor for the development of microalbuminuria over 5 years [20]. Analysis of 1441 people with type 1 diabetes in the Diabetes Control and Complications Trial (DCCT) cohorts suggests that systolic blood pressure levels below 120 mmHg are associated with a 41% reduction in macroalbuminuria (95% confidence interval [CI] 5 to 63%) and a 68% reduction in CKD stage 3 (95% CI 25 to 84%) [21]. The Pittsburgh EDC study 25-year follow-up results support an optimal blood pressure of 120/80 in childhood onset type 1 diabetes [22].

The threshold for diagnosis of hypertension in people with type 1 diabetes varies according to national and international guidelines. The Kidney Disease Improving Global Outcomes (KDIGO) guidelines suggest a blood pressure goal of ≤140/90 mmHg if urinary AER is under 30 mg per 24 h, or ≤ 130/80 mmHg if AER exceeds 30 mg per 24 h, although they do not distinguish between type 1 and type 2 diabetes [23]. National Institute for Health and Care Excellence (NICE) guidelines on the management of people with type 1 diabetes suggest a blood pressure target of 130/80 mmHg in someone with albuminuria (135/85 mmHg in those who were normoalbuminuric) [24]. The American Diabetes Association and American Society of Nephrology consensus guidelines suggest that a blood pressure of < 140/90 mmHg should be attained in all individuals with diabetes and renal disease, but they do not suggest a lower target and they do not distinguish between type 1 and type 2 diabetes [25]. In children with type 1 diabetes, the International Society for Pediatric and Adolescent Diabetes (ISPAD) defines hypertension as average systolic blood pressure and/or diastolic blood pressure that is greater than the 95th percentile for the person’s gender, age and height on more than three occasions, and suggests a target blood pressure of 130/80 mmHg [26]. Given the younger age of many adults with type 1 diabetes and the consequent longer lifetime blood pressure burden, we support the targets of 120–140/80 mmHg according to age and the presence or absence of albuminuria, with those aged over 65 being better suited to the 140/90 mmHg target [27–29].

The role of home and ambulatory blood pressure measurement in the diagnosis and management of hypertension in people with type 1 diabetes and nephropathy is unclear. Small cohort studies of children and adults with type 1 diabetes suggest that an increase in nocturnal systolic blood pressure or blunting of nocturnal dipping is an important factor in progression to microalbuminuria in people with type 1 diabetes [30, 31]. Due to a lack of robust evidence, no guidelines currently recommend ambulatory or home blood pressure monitoring to diagnose or manage hypertension in people with type 1 diabetes, although the ISPAD guidelines suggest that there may be a role for 24-h blood pressure monitoring in the diagnosis of hypertension in children [26].

The importance of lifestyle measures (weight loss and salt intake reduction) are highlighted by a number of guidelines, and indeed a recent study suggests that lower sodium intake may improve the efficacy of RAAS blockade [32].

There is evidence to suggest that management of blood pressure in people with type 1 diabetes may be suboptimal. In a large cross-sectional study of people with type 1 diabetes in Scandinavia, those on antihypertensive therapy who were achieving a blood pressure below 130/80 mmHg varied according to degree of albuminuria [33]. Blood pressure above 130/80 mmHg was seen in 74.6% of people who were normoalbuminuric; 71.2% of people who were microalbuminuric; 80.0% of people who were macroalbuminuric; 88.1% of people who were treated with dialysis; and 90.4% of people who had received a renal transplant.

An important point to consider is the presence of postural hypotension in people with type 1 diabetes. Autonomic neuropathy is often associated with postural hypotension, and people with type 1 diabetes should have their supine and standing blood pressure checked. A significant drop in blood pressure on standing (> 20 mmHg) might alert the clinician to ensure that care is taken not to treat the person’s blood pressure over-aggressively. Otherwise we advocate the use of upright (sitting or standing) blood pressure as the target blood pressure, as per British Hypertension Society guidelines [34].

#### Modulation of the RAAS in people with type 1 diabetes

##### Normoalbuminuria

There has been some interest in the use of agents that block the RAAS in the primary prevention of diabetic renal disease. The use of ACEIs has been tested in people who are normotensive and normoalbuminuric, and there is little evidence of a protective effect on the development of diabetic nephropathy. Importantly, however, many of these studies have used definitions of blood pressure that would now be considered to be too high. A multicentre European study examined 530 people with type 1 diabetes and blood pressure under 155/90 mmHg [35]. The study found that during 2 years of treatment with lisinopril versus placebo, the ACEI showed no protective effect against the development of microalbuminuria. Similar findings have been shown with candesartan [36]. Furthermore, a renal biopsy study of 285 people with type 1 diabetes who were normotensive and normoalbuminuric showed no effect of enalapril or losartan in the development of renal lesions [37]. One short study did suggest a significant reduction in urine albumin:creatinine ratio (UACR) in 89 individuals with type 1 diabetes who were normotensive and normoalbuminuric and who were treated with placebo or perindopril for 4 months [38]. Currently however, the use of ACE inhibition in people with type 1 diabetes who are normotensive and normoalbuminuric cannot be recommended on the basis of trial evidence.

##### Microalbuminuria

The onset of microalbuminuria in someone with type 1 diabetes was once thought to lead to inevitable progression to macroalbuminuria and thence to progressive kidney disease [39]. More recently, however, it has become clearer that microalbuminuria may remit in up to 40% of people with type 1 diabetes [40–42]. In addition, microalbuminuria may not progress to macroalbuminuria in a significant number of people [43]. In the Renin-Angiotensin System Study, onset of microalbuminuria correlated poorly with renal biopsy findings of diabetic glomerulopathy [44]. Previous studies have also described progressive renal impairment without microalbuminuria in people with type 1 diabetes [44, 45].

In adolescents with type 1 diabetes, modest but persistent elevations of UACR in the normal range may be associated with progression to persistent microalbuminuria [46]. Furthermore, a persistently raised UACR at the upper limit of the reference range in adolescents is associated with increasing aortic intima-media thickening, which is a sensitive marker of early atherosclerosis [47].

It is, however, recognised that the presence of microalbuminuria may not be the ideal risk marker for progressive renal dysfunction in people with type 1 diabetes [48]. Microalbuminuria may progress, stabilise or regress, and factors that govern this change are unclear, especially in adolescents and young adults who have improved glycaemia control [41]. Therefore, more reliable biomarkers or genetic markers are needed to predict which individuals are at the greatest risk of progressive renal disease. Many studies have looked at putative genetic loci within the RAAS for a genetic predisposition to diabetic nephropathy, but no clear correlation with nephropathy risk has been found in most studies [49]. Some authorities suggest that determination of serum cystatin C in people with diabetes and proteinuria may provide better risk stratification of subsequent end-stage kidney disease than determination of serum creatinine [50]. Serum concentration of tumour necrosis factor (TNF) receptors 1 or 2 (TNFR1, TNFR2) may also be predictors of future development of CKD stage 3 in people with type 1 diabetes [51]. If those who are at high risk of progression to diabetic nephropathy could be identified early, more intensive systematic therapy could be considered, for example closed loop insulin delivery system or pancreas transplantation [52].

There are few long-term studies that suggest that treating people with type 1 diabetes, microalbuminuria and normal blood pressure reduces end-stage kidney disease. There are, however, more short-term studies that focus on a change in AER rather than a change in renal function. A multicentre European study examined 79 individuals with microalbuminuria and blood pressure below 155/90 mmHg, and found a significant reduction in AER in the group of people who were treated with lisinopril compared with those who were treated with a placebo (− 34.2 mg/min) [17]. In an 8-year follow-up of a small number of people with type 1 diabetes and microalbuminuria, 10% of people who were treated with captopril progressed to macroalbuminuria, compared with 40% of those who were treated with a placebo [53]; therefore, treatment with captopril was associated with a reduction in progression of renal disease. Similarly, the Microalbuminuria Collaborative Study Group treated 235 people with microalbuminuria and blood pressure < 160/90 mmHg with placebo or captopril [54]. Progression to macroalbuminuria was seen in 21.9% of the placebo-treated group compared with 7.2% of the captopril-treated group (the risk reduction was 69%). The Ace-Inhibitor Trial to Lower Albuminuria in Normotensive Insulin-Dependent Subjects (ATLANTIS) study used ramipril versus placebo in 140 people with type 1 diabetes and microalbuminuria and normotension, and it showed regression to normoalbuminuria in 20% of the ramipril-treated group, compared with 4% of the placebo-treated group [55]. A further study of 20 individuals with type 1 diabetes, microalbuminuria and normal blood pressure who were treated with placebo or enalapril showed a reduction in progression to macroalbuminuria and a significant number of people regressed to normoalbuminuria [56].

RAAS blockade may have positive longer-term impacts on renal haemodynamics in people with type 1 diabetes even when therapy is stopped. In the 5-year Renin-Angiotensin System Study, people who were on RAAS blockade during the trial, but who stopped therapy after the trial, showed significantly greater renal haemodynamic responses to clamped hyperglycaemia and flow-mediated vasodilatation, which suggests that RAAS blockade has sustained, long-term protective effects [37].

In people who are hypertensive and have microalbuminuria, however, there is relatively strong evidence to suggest that ACE inhibition slows progression of diabetic nephropathy in people with type 1 diabetes and microalbuminuria [57]. Meta-analysis suggests that ACEIs reduce progression of microalbuminuria to macroalbuminuria (odds ratio 0.38; 95% CI 0.25 to 0.57) [58]. Outcomes in terms of the prevention of end-stage kidney disease, however, have not been reported.

##### Macroalbuminuria

For decades, the presence of macroalbuminuria in people with type 1 diabetes has been considered to be a stage of irreversible kidney disease. However, recent long-term follow-up of 159 individuals with type 1 diabetes in the Diabetes Control and Complications Trial / Epidemiology of Diabetes Interventions and Complications (DCCT/EDIC) study showed that 10 years after onset of macroalbuminuria, the cumulative incidence of reduction to microalbuminuria was 52% [43]. The cumulative incidence of CKD stage 3 (estimated glomerular filtration rate (eGFR) < 60 mL/min/1.73 m^2^) was 32%, and the cumulative incidence of end-stage kidney disease was 16% after 10 years, with better glucose and blood pressure control being the main factors associated with a lower risk of CKD progression. Therefore, while macroalbuminuria appears to be an important renal disease risk marker, it is far from inevitable that relentless progression to end-stage kidney disease will occur.

Seminal studies in the 1980s suggested that early aggressive antihypertensive therapy could reduce the rate of decline of renal function in people with diabetic nephropathy [59], and further studies of people with type 1 diabetes, hypertension and macroalbuminuria demonstrated the specific protective effects of ACEI drugs on progression of albuminuria and renal disease [60–62]. Meta-analysis of these studies suggest a long-term beneficial effect on preventing doubling of serum creatinine and development of end-stage kidney disease [63].

##### Use of other agents that modulate the RAAS

Candesartan has been studied in people with type 1 diabetes and diabetic retinopathy [64]. A beneficial effect of candesartan was seen in the protection of retinopathy; although in one study, the benefit of RAAS blockade was limited to people with poor glucose control (glycated haemoglobin (haemoglobin A1c) > 7.5%) [15, 64]. Studies using ARBs have not been widely reported in type 1 diabetes and nephropathy. In a small Danish study, losartan was seen to attenuate AER in people with type 1 diabetes [65]. In the Renin-Angiotensin System Study, however [37], use of losartan did not appear to protect people from developing microalbuminuria: indeed 17% of people on losartan developed microalbuminuria compared with 6% on a placebo and 4% on enalapril over 5 years.

It has been suggested that aldosterone escape during long-term RAAS blockade may be a mechanism by which ACE inhibition fails to prevent progressive renal disease in people with type 1 diabetes [66]. Thus, the use of aldosterone antagonists in such individuals may be useful. Spironolactone has been investigated in a small study of people with type 1 diabetes and microalbuminuria [67]. Spironolactone added to standard antihypertensive therapy reduced AER by 60%, with no drop in blood pressure and a minor drop in eGFR, although moderate hyperkalaemia was seen in a small number of individuals.

Aliskiren, the direct renin inhibitor, has been trialled in people with type 2 diabetes and diabetic nephropathy, and no significant effect on renal outcomes has been noted, although a reduction in AER has been noted [68]. A small study of people with type 1 diabetes who were treated with aliskiren showed positive effects on renal haemodynamic indices and systemic vascular responses [69]. Furthermore, dual blockade with ACEI also showed beneficial effects on arterial compliance, flow-mediated dilatation and renal vasodilatation [70]. Further study of this group of drugs in type 1 diabetes is warranted.

Early studies of beta-blockade in people with diabetic nephropathy and type 1 diabetes suggest an equivalent effect to ACEI [71]. There is some suggestion that non-dihydropyridine calcium channel blockade may have some of the benefits of dihydropyridine calcium channel blockers in the management of diabetic nephropathy [72].

Through their ability to reduce intraglomerular pressure, blood pressure and uric acid levels, sodium glucose cotransporter-2 (SGLT-2) inhibitors may offer the possibility of renal protection. One study suggests that SGLT-2 inhibitors can offer a reduction in glomerular hyperfiltration [73]. Recent analysis of the Empagliflozin, Cardiovascular Outcomes and Mortality in Type 2 Diabetes (EMPA-REG) study suggests significant renoprotection [74]. There is evidence from the Tandem 1 and 2 studies that sotagliflozin can reduce blood pressure and UACR over 12 months, with similar renal haemodynamic changes seen with SGLT-2 inhibition in type 2 diabetes [75]. However, the risk that these agents can cause ketoacidosis when they are given to people with type 1 diabetes may limit their potential use in this group [76].

##### Use of dual blockade in people with type 1 diabetes and diabetic nephropathy

Meta-analysis of a number of these studies of people with type 2 diabetes and nephropathy suggest a reduction in proteinuria, but at the expense of an increased risk of severe hyperkalaemia and episodes of acute kidney injury (AKI) [77–79]. More recently, however, a large randomised controlled trial involving people with type 2 diabetes suggests that RAAS dual blockade has no benefit in terms of mortality, but that it may increase the risk of hyperkalaemia and AKI [80].

It is currently unclear whether there is a role for dual blockade in people who have type 1 diabetes and a normal eGFR (> 60 mL/min/1.73 m^2^) in whom albuminuria is uncontrolled or increasing. While this may reduce albuminuria, there is no evidence of a reduction in other renal or cardiovascular end points.

In type 1 diabetes the pathogenic processes that occur in the development and progression of diabetic nephropathy may be very different. Use of ACEIs is associated with a compensatory increase in plasma renin activity, and this effect may be ameliorated by the use of ARB drugs. There are, however, few studies on the use of dual RAAS blockade and outcomes in type 1 diabetes. One small study from India of 30 people who were treated for a short period with telmisartan and ramipril resulted in a reduction in UACR and blood pressure, with a slightly increased risk of hyperkalaemia [81]. A further small study of 21 people with type 1 diabetes showed that the addition of irbesartan to ACEI therapy in people with type 1 diabetes resulted in a 37% reduction in AER, along with significant reductions in blood pressure [82]. Further studies of dual RAAS blockade in type 1 diabetes are needed.

##### When should RAAS blockade be stopped?

The use of RAAS-blocking drugs in early pregnancy has been associated with harm to the fetus, including cardiovascular, neurological and renal malformations [83], although more recent surveys do not suggest that there is a high risk of these problems occurring [84]. Pregnancy is associated with a high risk of progression of diabetic nephropathy in people with type 1 diabetes, and the benefits of RAAS blockade in such individuals may outweigh the risks, but current advice is that RAAS-blocking drugs should be stopped when pregnancy is confirmed, and indeed when pregnancy is planned.

Drugs that block the RAAS reduce intraglomerular pressure and may lead to a rise in serum creatinine of up to 30%, which should then stabilise [85]. Some studies suggest that clinically significant renal artery stenosis may be quite common among people with diabetes, especially those with type 2 diabetes [86]. While the use of drugs that modulate the RAAS may increase the risk of deterioration in renal function in people with renovascular disease, in practice such deterioration is rare [87, 88].

RAAS blockade can lead to hyperkalaemia, which may be managed by dietary methods, diuretics or use of sodium bicarbonate. However, if the hyperkalaemia is severe and refractory to these measures, RAAS blockade may need to be stopped or reduced but this needs balanced with the loss of the benefits of proteinuria reduction and retardation of GFR declin e[89]. A further possible clinical scenario is someone with type 1 diabetes having deteriorating renal function despite having well-controlled blood pressure on ACEI drugs. Once renal dysfunction continues to escape, despite optimal therapy, a decision may need to be made about cessation of ACEI therapy, especially if there may be a degree of ischaemic nephropathy, renovascular disease or postural hypotension. RAAS blockade may also increase the risk of AKI in people with diabetes, and advice to stop these drugs during periods of acute illness should be considered [90].

### Hypertension management and renin-angiotensin-aldosterone system blockade in people with type 2 diabetes, nephropathy and/or early CKD stages 1–3

#### Recommendations (Table 3)

Diabetic nephropathy is the leading cause of end-stage kidney disease and it is characterised by a triad of persistent albuminuria, hypertension and a decline in glomerular filtration rate (GFR). The presence of diabetic nephropathy increases cardiovascular morbidity and mortality and also increases progression to end-stage kidney disease [91–93]. After approximately 20–25 years, 40% of people with type 2 diabetes develop evidence of diabetic nephropathy [94]. Both hypertension and hyperglycaemia are strong risk factors in determining progression of end-stage kidney disease and cardiovascular complications in diabetic nephropathy. Microalbuminuria is one of the earliest manifestations of kidney disease in people with diabetes and it predicts increased cardiovascular morbidity and mortality in people with both type 1 and type 2 diabetes [95, 96]. The prevalence of microalbuminuria in people who have had type 2 diabetes for 10 years is 25%, with an annual rate of progression to overt nephropathy of approximately 3% [94].

**Table 3:** Recommendations for people with type 2 diabetes and early CKD stages 1–3

Recommendations for renin-angiotensin-aldosterone system (RAAS) blockade and hypertension management in people with type 2 diabetes, nephropathy and/or early chronic kidney disease (CKD)	
1. In people with type 2 diabetes and hypertension, we recommend salt intake of < 90 mmol per day (< 2 g per day of sodium – equivalent to 5 g of sodium chloride) (Grade 1C). 2. In people with type 2 diabetes, CKD and urine albumin:creatinine ratio (UACR) < 3 mg/mmol (< 26.55 mg/g), we recommend that their target upright blood pressure should be < 140/90 mmHg, using antihypertensive therapy in the maximum tolerated doses (Grade 1D). 3. In people with type 2 diabetes, CKD and UACR of > 3 mg/mmol (> 26.55 mg/g), we suggest aiming for a target upright blood pressure that is consistently < 130/80 mmHg, using antihypertensive therapy in the maximum tolerated doses (Grade 2D). 4. There is no evidence to support either ACEI or ARB therapy as first-line blood pressure lowering agents in comparison with other antihypertensive agents in people with type 2 diabetes, normal renal function and normal UACR (< 3 mg/mmol [< 26.55 mg/g]) (Grade 1A). 5. We suggest that ACEIs (or ARBs if ACEIs are not tolerated) should be preferentially used in people with type 2 diabetes and CKD who have UACR > 3 mg/mmol (> 26.55 mg/g). We recommend that the dose of ACEI (or ARB) should be titrated to the maximum tolerated (Grade 2D). 6. There is currently no evidence to support the role of home or ambulatory blood pressure monitoring in people with type 2 diabetes and CKD stages 2 and 3 (Grade 1D). 7. There is currently no evidence to support the role of dual blockade of the RAAS in people with type 2 diabetes and CKD stages 1 to 3 (Grade 1B). 8. Upright blood pressure targets should be set at no lower than 150/90 mmHg in those with type 2 diabetes who are aged 75 years or over (Grade 2B). 9. We recommend that people with type 2 diabetes should be advised to stop RAAS-blocking drugs during periods of acute illness and restarted 24–48 h after recovery from the illness (Grade 1C).	

The risk of new as well as progressive microalbuminuria is significantly associated with high blood pressure [97]. In people with diabetes, cardiovascular and renal outcomes are adversely affected by the presence of hypertension and albuminuria [98]. Thus, controlling blood pressure and reducing albuminuria are important treatment goals in diabetic nephropathy. Baseline blood pressure levels have been shown to be a powerful determinant of subsequent kidney failure in large population-based studies [99, 100]. Unlike those with type 1 diabetes, a high proportion of people with type 2 diabetes often have microalbuminuria and overt nephropathy at diagnosis. Without intervention, 20–40% of people with type 2 diabetes and microalbuminuria will progress to overt nephropathy. After 20 years of overt nephropathy, approximately 20% of those people will progress to end-stage kidney disease.

#### The renin-angiotensin-aldosterone system

Dysregulation of the renin-angiotensin-aldosterone system (RAAS) plays a vital role in the pathogenesis of diabetic nephropathy, including pathogenesis of both micro- and macrovascular complications. Hyperglycaemia is associated with increased production of angiotensin II following RAAS over-activation in glomerular mesangial cells [101]. Thus, mechanisms to block the RAAS are an important therapeutic target in people with type 2 diabetes and nephropathy.

#### Hypertension in people with type 2 diabetes

In nearly one-third of people with type 2 diabetes, hypertension is present at the time of their diagnosis. Hypertension and type 2 diabetes may be related to underlying diabetic nephropathy, to co-existing essential hypertension or to renovascular disease, or it may be part of the complex insulin resistance syndrome. Hypertension in people with type 2 diabetes is generally associated with expanded plasma volume, increased peripheral vascular resistance and low renin activity [102].

The threshold for diagnosis and aims for hypertension control in people with type 2 diabetes vary according to national and international guidelines. In the UK, for the management of hypertension in people with diabetes and for those with a UACR of 70 mg/mmol (619.47 mg/g) or more and CKD, NICE guidance recommends a target blood pressure of < 130/80 mmHg [34]. The American Diabetes Association and the American Society of Nephrology recommend a blood pressure of < 140/90 mmHg in all individuals with type 2 diabetes and renal disease [103]. The Kidney Disease Improving Global Outcomes (KDIGO) guidelines recommend a blood pressure of ≤140/90 mmHg in those who have an AER of < 30 mg per 24 h (UACR > 3 mg/mmol [> 26.55 mg/g]), or ≤ 130/80 mmHg if the AER is > 30 mg per 24 h (UACR > 3 mg/mmol [> 26.55 mg/g]) in people with type 2 diabetes [23].

The KDIGO 2020 guidance suggests a stricter control in all individuals with CKD targeting a systolic blood pressure < 120 mmHg using a standardised blood pressure measurement technique, but acknowledges the lack of evidence in people who have diabetes and CKD [23].

There is little evidence base for recommending blood pressure targets in older people who have CKD. Most randomised controlled trials excluded people who were over 70 years of age (mean age 65 years: about 2.5% were older than 85 years of age) but some indirect inferences can be drawn from studies of older populations who do not specifically have CKD. While there is some evidence regarding the treatment of high blood pressure in much older people (that is, older than 80 years of age) from the Hypertension in the Very Elderly Trial (HYVET) [104], it applies to a blood pressure target of 150/80 mmHg in people with CKD who have an eGFR > 40 mL/min/1.73 m^2^.

The Swedish Trial in Old Patients with Hypertension (STOP Hypertension), which compared antihypertensive treatments in preventing cardiovascular events in older people with diabetes (with a mean age of 75.8), supports an upright blood pressure target of no lower than 150/90 mmHg [105].

The KDIGO guidelines [23] suggest tailoring blood pressure treatment in older people with CKD to consider age, comorbidities and other therapies, with a gradual escalation of treatment and close attention to electrolyte disorders, acute deterioration in kidney function, orthostatic hypotension and side effects of medications. Thus, it would seem reasonable to suggest a target upright systolic blood pressure of no less than 150 mmHg in people with diabetes and CKD aged over 75, taking into account side effects of medications and comorbidities.

#### The role of home and ambulatory blood pressure measurement

Although home and ambulatory blood pressure monitoring is thought to be more representative of real-life blood pressure, their exact role in the diagnosis and management of hypertension is unclear because a limited number of studies have been conducted in people with type 2 diabetes who have CKD. However, it is recognised that high ambulatory blood pressure measurement systolic pressures and nocturnal non-dipping are associated with increased mortality and a decline in eGFR [106–108]. A small study of ambulatory blood pressure measurement in people with CKD, where 436 people who were hypertensive were prospectively followed up, showed that it was much more accurate in predicting both renal and cardiovascular outcomes than office blood pressure [109]. Self blood pressure monitoring and ambulatory blood pressure measurement utilises oscillometric assessment of blood pressure at the elbow, which may be influenced by irregularities of pulse and high pulse pressures. In the UK, NICE guidelines recommend confirming hypertension with 24-h ambulatory monitoring (home BP monitoring where 24-h BP monitoring is unsuitable or not tolerated) before starting or increasing antihypertensive agents [34]. However, there is no direct evidence supporting the use of either method for diagnosis of hypertension in people with type 2 diabetes and CKD.

#### Lifestyle modification and impact on blood pressure

There is good evidence from a number of observational studies and randomised controlled trials that salt intake, weight and body mass index (BMI), exercise frequency and alcohol intake all have a significant impact on blood pressure levels [111–114]. Please see Table 4 for details.

**Table 4:** Lifestyle modification and impact on blood pressure

**Salt intake**	
The evidence base for the benefit of salt restriction in type 1 diabetes without advanced CKD is not strong. Reduced blood pressure has been found in some but not all short-term studies, but an important long-term observational study recorded higher dietary sodium intake was associated with higher all-cause mortality and the development of ESKD [115, 116]. The KDIGO guidelines suggest lowering salt intake to < 90 mmol of sodium per day (< 2 g of sodium, which corresponds to 5 g of sodium chloride) [23]. High salt intake has a greater impact on blood pressure for people with diabetes, especially in those with CKD, due to their reduced ability to excrete salt load in their urine. Restricting salt intake lowers blood pressure by a moderate amount, as shown in a systemic review of seven trials where salt intake was restricted to 4–6 g (70–100 mmol), systolic blood pressure was reduced by 4.7 mmHg and diastolic blood pressure was reduced by 2.5 mmHg [117]. Given that salt restriction is inexpensive and it helps to lower blood pressure in the general population, despite a lack of availability of large-scale, long-term randomised controlled trials of salt restriction in people with CKD, there is no reason to believe that it would not be beneficial, although it would add to the dietary restrictions for managing diabetes. A low-salt diet has been shown to reduce blood pressure and albuminuria in the short term in people who are on angiotensin receptor blockers (ARBs) and it may be a consideration for those with high blood pressure who have had a poor response to ACEIs or ARBs [118, 119].	
**Weight and BMI**	
Although abdominal obesity has been associated with higher blood pressure and use of antihypertensive therapy in type 1 diabetes [120], there is a dearth of evidence that weight reduction in type 1 diabetes reduces blood pressure, although this would be expected intuitively [121]. There is evidence of weight gain accompanied by increases in blood pressure in type 1 diabetes as a consequence of improved blood glucose control. The KDIGO guidelines recommend achieving or maintaining a healthy weight (BMI 20–25) [23]. Some observational studies, but not randomised trials, suggest that weight loss is likely to improve blood pressure in people with CKD, but there is a lack of high-quality randomised controlled trials in this area. Although obesity has been proposed to be a potential mediator of CKD progression, trials are conflicting and reliable data remain sparse. There is no role of weight loss diets in CKD either. Overall, achieving a healthy body weight will improve blood pressure levels and prognosis in CKD, particularly in the early stages (stages 1–2). Malnutrition needs to be avoided in more advanced stages of CKD [122].	
**Exercise programme**	
There is documentation that exercise training for 12 weeks or more reduces blood pressure in type 1 diabetes [123]. The KDIGO guidelines recommend undertaking an exercise programme that is compatible with cardiovascular health and tolerance, aiming for at least 30 min of exercise five times per week [23]. Increased physical exercise has a broad range of positive health outcomes in the general population. However, there are no randomised controlled trials in the CKD population: there are mostly observation studies. The benefits of exercise on blood pressure and on general health are likely to be similar in the CKD population as they are in the general population [124].	
**Alcohol intake**	
Evidence that alcohol intake affects blood pressure and reduction in intake helps blood pressure in type 1 diabetes is sparse. The KDIGO guidelines suggest limiting alcohol intake to no more than two standard drinks per day for men and no more than one standard drink per day for women [23]. Most of the effects of alcohol reduction are related to its effect on blood pressure; that is, suggesting that restricting alcohol intake would lower blood pressure. All the trial evidence is mostly related to the general population and there are no specific data on people with CKD, but the effects of alcohol intake on blood pressure are expected to be similar [125].	

There is good evidence from a number of observational studies and randomised controlled trials that salt intake, weight and body mass index (BMI), exercise frequency and alcohol intake all have a significant impact on blood pressure levels [111–114]

#### Blood pressure lowering agents

**Fig. 2:**
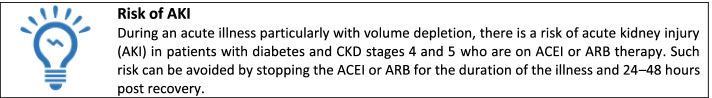
Sick day rule

In people with type 2 diabetes and CKD, three or more blood pressure agents are frequently required. There is increasing emphasis on individualisation of therapy. Eventually, the choice of agent is less important than the actual reduction in blood pressure that is achieved. There is little evidence to support the use of any particular agent in controlling blood pressure in CKD, nor are there any data to suggest the choice of second- or third-line medications. The exception to this rule is the use of ACEIs or ARBs in people with CKD who have proteinuria. ACEIs and ARBs have each been shown to be effective in delaying disease progression in people with type 2 diabetes who have microalbuminuria or established diabetic nephropathy. There is a need to escalate to the maximal doses of ACEI or ARB in people who have diabetes and albuminuria before moving on to additional agents in order to achieve the required blood pressure targets. However, there is no evidence that these agents are effective in the primary prevention of diabetic nephropathy. The use of ACEIs or ARBs in people with type 2 diabetes reduces microalbuminuria and retards the progressive loss of renal function [61, 126–128]. ARBs are said to provide renoprotection over and above their blood pressure lowering effect and short-term albuminuria reduction, and they are said to have a long-term favourable effect on renal prognosis [129] (Fig. 2).

#### Adherence with therapy

Non-adherence to antihypertensive treatment is common, with over 50% of people with apparent treatment resistance being non-adherent. This is especially so in those who are on multiple anti-hypertensive and other medications. Urine antihypertensive drug monitoring may help management of these individuals [130]. Therefore, it is important to assess adherence especially in those not achieving blood pressure control despite taking 3 or more antihypertensive agents in optimum doses.

#### RAAS blockade in people with type 2 diabetes without proteinuria

The use of RAAS blockade has significant benefits on cardiovascular and renal end points in people with diabetes, independent of their blood pressure lowering effect, as shown in the Heart Outcomes Prevention Evaluation (HOPE) trial and the European trial on reduction of cardiac events with perindopril in stable coronary artery disease [131, 132]. Whereas most guidelines favour the use of RAAS blockade as first-line treatment for people with diabetes, hypertension and CKD (the American Diabetes Association, the American Society of Hypertension, the International Society of Hypertension) [133, 134], the European Society of Cardiology / European Society of Hypertension guidelines from 2013 and the eighth Joint National Committee on Prevention, Detection, Evaluation and Treatment of High Blood Pressure from 2014 recommend the use of *any* class of antihypertensive agent in people with diabetes in the *absence* of proteinuria, but suggest the use of RAAS blockade as first-line treatment *only* in the presence of proteinuria [135, 136].

This is based on the findings of 19 randomised controlled trials that enrolled 25,414 participants with diabetes, with a total of 95,910 patient years of follow-up. The results of this study from head-to-head randomised trials of RAAS blockade versus other antihypertensive agents failed to show superiority of RAAS blockade in people with diabetes and *no proteinuria*, and it suggested that any class of antihypertensive agents can be used in such individuals [137].

#### RAAS blockade in people with type 2 diabetes and proteinuria or microalbuminuria

In the UK, NICE guidance suggests offering a low-cost RAAS antagonist to people with CKD and diabetes who have: a UACR of 3 mg/mmol (26.55 mg/g) or more; hypertension and a UACR of 30 mg/mmol (265.49 mg/g) or more; or a UACR of 70 mg/mmol (619.47 mg/g) or more irrespective of hypertension or cardiovascular disease [138]. The favourable effects of RAAS blockade have been seen mainly in placebo-controlled trials [128, 131] and it has been postulated that the benefits of RAAS blockade on renal outcomes was probably as a result of their blood pressure lowering effect [139]. Several major trials have also demonstrated clear benefits of ARB use in people who have diabetic nephropathy [140, 141].

#### Use of dual blockade with ACEIs and ARBs in people with type 2 diabetic nephropathy

ACEIs and ARBs block the RAAS at different sites and, in theory, dual blockade should provide more effective and complete blockade of the RAAS. The rationale for dual blockade is based on a phenomenon called ‘angiotensin II escape’, whereby evidence suggests that standard doses of ACEIs only offer a partial blockade of the angiotensin-converting enzyme (ACE) [142]. It is said that enzymes such as chymase and cathepsin G can generate angiotensin II from angiotensinogen and other peptide substrates [143].

Several early studies had suggested that using a combination of ACEI/ARB provided additional benefit in diabetic nephropathy in terms of surrogate albuminuria lowering. However, there remains substantial controversy about whether ACEIs and ARBs should be combined, given that most of these studies were small in size and short in duration.

In one meta-analysis of 10 trials, 156 participants received a combination of ACEI/ARB and 159 received an ACEI only (the duration of the study was 8–12 weeks). The combination was shown to reduce proteinuria at the expense of statistically and clinically significant reductions in eGFR. There was a suggestion that this decrease could be secondary to a reduction in blood pressure alone [77]. Most of the evidence base for combination dual blockade therapy initially came from studies about heart failure without any long-term data to support it (the candesartan and lisinopril microalbuminuria (CALM) study) [144]. This study evaluated the effects of dual blockade of candesartan and lisinopril on blood pressure and microalbuminuria in 199 people with type 2 diabetes (the duration of the study was 24 weeks). At the end of the study, combination therapy was found to be significantly more effective in reducing UACR (50% with combination, 24% with candesartan and 39% with lisinopril) and diastolic blood pressure (16.3 mmHg, 10.4 mmHg and 10.7 mmHg reduction, respectively) than either agent alone. Criticisms of some of these studies were that there were no long-term follow-up data and that maximal doses of ACEIs were not used. It is also questionable whether the effects were specifically related to combination therapy or whether it was blood pressure reduction per se that was instrumental.

The ONTARGET study involved telmisartan and ramipril, and showed that the primary renal outcome (ie dialysis, doubling of serum creatinine and death) was similar for telmisartan (13.4%) and ramipril (13.5%), but was increased with combination therapy (14.5%, *p* = 0.037). The combination therapy, although associated with reduced albuminuria, caused the greatest decline in eGFR [145].

The KDIGO guidelines provide specific advice on dual blockade [23, 146]. In the UK, a NICE guideline explicitly states that combination therapy should not be used. The European Renal Best Practice working group has the same viewpoint as NICE [147]. The Canadian Health Education Programme’s (CHEP’s) 2009 recommendation advised against the use of dual blockade for people with non-proteinuric CKD or in people with diabetes and normal urinary albumin levels [148].

Overall, therefore, there is no current evidence to suggest a beneficial effect of ACEI/ARB combination on the progression of diabetic nephropathy. Instead, combination therapy resulted in clinically significant decreases in eGFR and hyperkalaemia [149]. In one meta-analysis involving 17,337 people, the adverse effects of dual blockade revealed significantly high rates of discontinuation because of a worsening of renal function, hyperkalaemia and symptomatic hypotension [150].

It is to be noted, however, that most studies published so far with regard to dual blockade have involved people with *normal renal function* who did not have a clinically significant rise in serum potassium or creatinine with dual blockade. However, in real life, widespread use of these agents would most likely involve people with resistant hypertension with chronic renal impairment, and such individuals therefore will tend to have more of these side effects.

#### Aldosterone blockade in people with type 2 diabetic nephropathy

Aldosterone, the principal physiological mineralocorticoid, has deleterious effects on both the cardiovascular system and the kidneys. There is evidence to suggest that initial RAAS blockade suppresses aldosterone levels. However, due to the phenomenon of aldosterone escape, aldosterone levels rise subsequently and can often exceed the baseline. ACEIs or ARBs do not directly block the effects of aldosterone at the receptor level [151].

Most evidence of the use of aldosterone antagonists like spironolactone, eplerenone and (more recently) finerenone come from heart failure trials. In one study of people who had type 2 diabetes with early nephropathy and normal renal function, adding spironolactone to ACEI treatment was shown to be clinically useful and safe for people who showed aldosterone escape during ACEI treatment and who no longer showed maximal antiproteinuric effects of ACE inhibition [152, 153]. In another study of people with type 2 diabetes, macroalbuminuria and serum creatinine of less than 160 μmol/l, treatment with spironolactone was found to be superior to cilazapril in reducing albuminuria [154]. In that study, 50 mg of spironolactone was used and blood pressure of < 135/85 mmHg was pre-treated with atenolol and hydrochlorothiazide before randomisation. The authors concluded that the superior effect of spironolactone was independent of its hypotensive effect, although 15% of people had to discontinue spironolactone because of hyperkalaemia. However, since then this study publication has been retracted.

There is also evidence for additive effects of eplerenone (an aldosterone antagonist that does not have the oestrogenic side effects of spironolactone) like in the other aldosterone antagonist trials. Eplerenone was found to have beneficial effects on microalbuminuria in people with type 2 diabetes when it was added to enalapril, although there was a much higher incidence of hyperkalaemia [155]. In the largest randomised controlled trial available using eplerenone, people with CKD, elevated urinary albumin levels and type 2 diabetes (177 people) received 50–100 mg of eplerenone and 91 people received a placebo. The addition of eplerenone to enalapril 20 mg per day resulted in a 40–50% reduction in AER by 12 weeks in the eplerenone group, but by less than 10% in the placebo group. Small reductions in eGFR and systolic blood pressure were noted, as was hyperkalaemia [156].

More recently, finerenone, a novel non-steroidal mineralocorticoid antagonist with greater receptor selectivity than spironolactone and eplerenone, has been shown to provide a greater reduction in proteinuria and end organ damage, compared with spironolactone or eplerenone. This was shown in the Mineralocorticoid Receptor Antagonist Tolerability – diabetic nephropathy (ARTS-DN) study involving 1501 participants who were already receiving an ACEI or ARB (the mean age of the participants was 64.2 years, 37% had a UACR > 30 mg/mmol [> 265.49 mg/g] and 40% had an eGFR of 60 mL/min/1.73 m^2^ or lower). Finerenone reduced the UACR at day 90 (relative to the baseline) more significantly than the placebo, and the pre-specified secondary outcome of hyperkalaemia leading to discontinuation was not observed either in the placebo or the finerenone group at various dosages. Also, there was no difference in terms of the incidence of a greater than 30% decrease of eGFR in either group. Thus 2.5–10 mg finerenone per day reduced albuminuria from the baseline in individuals with CKD and heart failure with a lower incidence of hyperkalaemia than spironolactone. It was postulated that this new mineralocorticoid receptor antagonist may be able to address the unmet medical need of safely managing albuminuria without effecting serum potassium in people with type 2 diabetes who have nephropathy. The strength of the study is that there was only a modest reduction in blood pressure at the highest dose of finerenone: quite unlike any other mineralocorticoid antagonist study in the past. The limitations of the study, however, include its short duration, the lack of a control group and that 60% of participants had an eGFR above 60 mL/min/1.73 m^2^, which put them at relatively low risk of hyperkalaemia [157].

The FIDELIO-DKD study showed that finerenone, a non-steroidal selective mineralocorticoid receptor antagonist, lowered the risks of CKD progression and cardiovascular events in people with CKD and type 2 diabetes treated optimally with renin-angiotensin system blockade. Of the 5734 people in the study, over 54% had eGFR < 45 mL/min/1.73 m^2^ and the median albumin creatinine ratio was 852 [IQR: 446–1634]. Finerenone, in doses of 10–20 mg daily, was associated with improved renal (HR 0.82; 95% CI 0.73 to 0.93; NNT 29) and cardiovascular outcomes (HR 0.86; 95% CI 0.75 to 0.99; NNT 42) compared with placebo. In the study cohort, finerenone reduced systolic blood pressure by 3 mmHg and the incidence of hyperkalaemia of > 6 mmol/L was 10% with discontinuation due to serious hyperkalaemia of 2.5% [158, 159].

From the above evidence, it may be reasonable in the future to consider adding in a selective mineralocorticoid receptor antagonist in people with DKD with a serum potassium of < 5 mmol with worsening albuminuria who are already on a maximal dose of ACEI or ARB.

#### Use of direct renin inhibitors in diabetic nephropathy

The use of aliskiren in people with type 2 diabetes and nephropathy has been shown to reduce AER, although no significant effects on renal outcomes have been noted. In the ALTITUDE trial, where aliskiren or a matching placebo was used on top of an ACEI or ARB in people with diabetic nephropathy, there were significant reductions in proteinuria but the trial was stopped early due to the low likelihood of ever demonstrating a benefit and the suggestion of an increased risk of non-fatal stroke, renal complications, hyperkalaemia and hypotension [160]. The drug has subsequently been withdrawn from the market.

In another trial involving 599 participants, aliskiren was used either alone or in combination with losartan for 6 months. This resulted in a reduction of UACR by 20% compared with the use of losartan alone. There were small differences in blood pressure between the two groups but no difference was found between the rates of adverse events [161]. Direct renin inhibitors are not currently recommended for use in diabetic nephropathy.

#### When should RAAS blockade be stopped?

Although ACEIs and ARBs are valuable blood pressure lowering agents in people with type 2 diabetes and CKD, they are not without their side effects.

##### Hyperkalaemia

In the UK, NICE guidance suggests measuring serum potassium and eGFR before starting RAAS blockade and repeating the measurements 1–2 weeks after starting RAAS blockade and after each dose increase. NICE further says not to offer these agents if the person’s pre-treatment serum potassium is > 5 mmol/L [34]. NICE guidance also suggests that these agents should be stopped if the serum potassium concentration increases to 6 mmol/L or more, and other drugs known to promote hyperkalaemia have been discontinued. However, recent NICE technology appraisals recommend the use of potassium binders, patiromer and sodium zirconium cyclosilicate, in outpatient care for people with persistent hyperkalaemia (≥6 mmol/L) and CKD stages 3b to 5 (non-dialysis), who are not taking an optimum dose of RAAS inhibitor because of hyperkalaemia [162, 163].

##### A drop in eGFR or an increase in serum creatinine

Given the basic pathophysiological mechanism of RAAS blockade, these agents cause a reduction in eGFR and urinary albumin excretion through efferent and afferent glomerular arteriolar dilatation, with a resultant fall in intra-glomerular blood pressure. A reversible reduction of eGFR of up to 30% can be expected. Greater reductions may indicate underlying renal artery stenosis.

NICE guidance states that if there is a decrease in eGFR of > 25% or an increase in serum creatinine of > 30% with RAAS blockade, renal function tests need to be repeated within 1–2 weeks. If the eGFR drops by 25% or more, or there is a change in serum creatinine by 30% or more, NICE guidance suggests conducting further investigations to identify a cause of renal deterioration, such as sepsis, volume depletion, other acute illneses such as heart failure and myocardial infarction, or non-steroidal inhibitor / potassium-sparing diuretic use. If no other cause for the deterioration in renal function is found, it is recommended to stop the RAAS blockade or reduce the dose to a previously tolerated lower dose, and add an alternative antihypertensive medication if required [138, 164, 165].

Consideration should also be taken where someone’s baseline eGFR is already below 30, especially in those with congestive cardiac failure, where there may be a broader benefit terms of left ventricular function.

##### Pregnancy

Given the potentially teratogenic nature of RAAS blockade drugs, the KDIGO guidelines suggest that the use of these drugs in women of childbearing age should be balanced with the risk of pregnancy [166].

##### Inter-current illness

There are risks of large reductions in eGFR with RAAS blockade, particularly during intercurrent illness or with intravascular fluid depletion (diarrhoea, vomiting and high fever). It is therefore recommended to reduce the dose or to hold off ACEI or ARB use until recovery is made, because ensuing hypotension may cause an acute decline in eGFR in people with type 2 diabetes with CKD who are taking ACEIs or ARBs. It is recommended that people with type 2 diabetes should be advised to stop RAAS-blocking drugs during periods of acute illness and restarted 24–48 h after recovery from the illness. These precautions should especially be taken if an individual is on a combination involving non-steroidal anti-inflammatory drugs or diuretics [167–170].

#### Other agents for blood pressure lowering in people with type 2 diabetes and nephropathy

Most of the evidence for the use of other antihypertensive agents (apart from ACEIs or ARBs) is extrapolated from the general population and there is little evidence of their specific use or rationale in people with type 2 diabetes and CKD.

##### Calcium channel blockers

There is good evidence to suggest that non-dihydropyridine calcium channel blockers (verapamil and diltiazem) reduce proteinuria [72, 171]. A multicentre trial in people with type 2 diabetes and nephropathy suggested that adding a non-dihydropyridine calcium channel blocker to an ACEI-based regime can be effective at lowering residual albuminuria with or without a significant reduction in systolic blood pressure [172]. Thus non-dihydropyridine calcium channel blockers can be used as a valid additive or alternative to ACEIs or ARBs in people with type 2 diabetes, suggesting that their renal protective effects are over and above blood pressure lowering alone. Diltiazem and verapamil can induce bradycardia and heart block in combination with beta blockers; dihydropyridines such as amlodipine are more appropriate alongside beta blocker use.

##### Beta blockers

Much of the bad publicity about beta blockers is related to the use of atenolol, which has been the most frequent comparator in most randomised controlled trials. However, beta blockers are not a homogenous class of drug, and agents like celiprolol, carvedilol and nebivolol have vasodilating properties and do not share the negative properties of atenolol (that is, a lack of 24-h antihypertensive effect and withdrawal effects). In the UK, NICE guidance does not favour beta blockers as the first-line choice in the treatment of hypertension in the general population. There is evidence that in people with type 2 diabetes with advanced CKD and a high risk of sudden death, beta blockers may prove to be beneficial by lowering heart rate apart from lowering sympathetic hyperactivity and preventing ventricular arrhythmias [173, 174]. A meta-analysis of beta blockers used to treat CKD supports the use of beta blockers in people with CKD who have heart failure, but it does not provide evidence of their efficacy in preventing mortality, cardiovascular events or renal disease progression in people with CKD who do not have heart failure [175].

##### Diuretics

In the UK, NICE guidance prefers agents with a thiazide-like action such as chlorthalidone and indapamide, and this is relevant for individuals with CKD who have type 2 diabetes [34]. Chlorthalidone was used in the largest randomised controlled trial in hypertension (the Anti-hypertensive and Lipid-Lowering Treatment to Prevent Heart Attack Trial (ALLHAT) study) [176]. The evidence base for indapamide is through the Hypertension in the Very Elderly Trial (HYVET) [104]. The PROGRESS trial involved a combination of indapamide and perindopril, and was shown to reduce the risk of stroke [177]. The additional advantage of indapamide is its potassium-depleting effect, and this may be convenient when it is combined with ACEIs or ARBs, particularly in those with type 2 diabetes who have CKD. Loop diuretics like furosemide would be particularly useful for treatment of hypertension in people with type 2 diabetes with advanced CKD stages 4–5, as fluid overload is invariably a major contributing factor in such individuals.

##### Alpha blockers

Drugs like doxazosin could be an adjunctive treatment for hypertension in people with type 2 diabetes and CKD in whom other therapies have failed or not been tolerated, particularly if symptoms of prostatic hypertrophy are present. Alpha blockers are generally not recommended first line because of the common side effects of postural hypotension, tachycardia and headache.

##### Centrally acting alpha adrenergic agonists

Centrally acting alpha adrenergic agonists cause vasodilation by reducing sympathetic outflow from the brain. Common agents in this category are methyldopa, clonidine and moxonidine. Doses of methyldopa and clonidine are not generally required to be reduced in people with CKD. Although moxonidine is extensively excreted by the kidney, one randomised controlled trial that compare it with a calcium channel blocker added to an ACEI or ARB plus a loop diuretic indicated that it is safe to be used in advanced CKD [178]. Common side effects of moxonidine include headache, tiredness, dizziness and gastrointestinal symptoms, which occur in 10–15% of people. These agents should not be used as a first-line treatment, but they are generally used in conjunction with other antihypertensive agents in people with type 2 diabetes who have hypertension.

#### Agents shown to have benefit in blood pressure reduction and outcomes but not currently licensed for this indication

##### Endothelin a receptor antagonists

Atrasentan, an endothelin A receptor antagonist, 0.75 mg daily, was able to reduce systolic blood pressure significantly in 2648 people who were proteinuric with diabetes and CKD and with eGFR 25–75 mL/min/1.73 m^2^. They were responsive to the drug by 6.1 mmHg (95% CI 5.6 to 6.7) and by 1.2 mmHg (95% CI 0.7–1.7) in the subsequent RCT which led to lower the risk of ESKD and doubling of serum creatinine by 35%; HR 0.65 (95% CI 0.49–0.88). Therefore, endothelin A receptor antagonists are promising agents in lowering BP but further studies in high-risk advanced CKD individuals are necessary before clinical use [179].

##### Sodium glucose cotransporter-2 (SGLT-2) inhibitors

In the CREDENCE trial systolic blood pressures were lower in the canagliflozin group by 3.30 mmHg (95% CI 2.73 to 3.87) and diastolic blood pressure by 0.95 mmHg (95% CI, 0.69–0.92) compared with the placebo group.178 Despite the greater reduction in eGFR in the first 3 weeks in the canagliflozin group (− 3.17 mL/min/1.73 m^2^, 95% CI, − 3.87 to − 2.47) the longer term decline in kidney function was slower in the canagliflozin group by 2.74 mL/min/1.73 m^2^ per year (95% CI, 2.37, 3.11). This phenomenon is very similar to what is seen with RAASi and hence SGLT-2i may have a similar mechanism of action. There was also a 31% reduction of UACR in the canagliflozin group. The majority of the participants had CKD stage 3 with mean eGFR at baseline of 56.2 ± 18.2 mL/min/1.73 m^2^ with significant albuminuria 300–5000 mg/g. However, 373 people reached the study endpoints of end-stage kidney disease, doubling of serum creatinine and renal death indicating a group of people with CKD stages 4 and 5 benefited from continued canagliflozin even at eGFRs < 30 mL/min/1.73 m^2^ [180, 181].

### Hypertension management and renin-angiotensin-aldosterone system blockade in people with type 2 diabetes, nephropathy and/or later stage CKD stages 4 and 5 (non-dialysis)

#### Recommendations (Table 5)

Advanced stages of CKD, particularly stages 4 and 5, are associated with hyperkalaemia, fluid retention and anaemia requiring erythropoiesis stimulating agents which may further increase blood pressure. Hyperkalaemia [> 5.5 mmol/L] is present in 31% of people in advanced kidney disease clinics [182]. Hyperkalaemia is more common in people with CKD and diabetes than in those with CKD without diabetes [183]. In a blood pressure control trial in people with CKD the risk of hyperkalaemia was seven times higher in people with eGFR < 30 mL/min/1.73 m^2^ compared with eGFR > 50 mL/min/1.73 m^2^ and seven times higher with ramipril compared with amlodipine [184]. Hence BP control in people with diabetes and CKD stages 3–5 particularly with an ACEI or ARB requires careful monitoring and management of serum potassium. The prevalence of primary aldosteronism in people with diabetes and resistant hypertension is 14%, and this should be considered when BP is difficult to control [185].

**Table 5:** Recommendations for people with type 2 diabetes and CKD stages 4 and 5 (non-dialysis)

Recommendations for hypertension management and RAAS blockade in people with type 2 diabetes and CKD stages 4 and 5 (non-dialysis)	
1. We recommend regular monitoring of blood pressure, urine albumin, blood electrolytes and kidney function in people with diabetes and CKD stages 4 and 5 (Grade 1B). 2. We suggest, if blood pressure is uncontrolled, electrolytes are abnormal, or kidney disease is progressive they should be monitored 2 to 4 times a year depending on the stage of CKD and the individual’s need (Grade 1B). 3. We recommend initiation of antihypertensive agents in people with diabetes and CKD stages 4 and 5, and UACR < 3 mg/mmol (< 26.55 mg/g) when blood pressure is ≥140/90 mmHg and aim for a target blood pressure of < 140/90 mmHg during therapy (Grade 1B). 4. We suggest initiation of antihypertensive agents in people with diabetes and CKD stages 4 and 5 and UACR > 3 mg/mmol (> 26.55 mg/g) when blood pressure is ≥130/80 mmHg and aim for a target blood pressure < 130/80 mmHg (Grade 2C). 5. We recommend the use of angiotensin converting enzyme inhibitor (ACEI) (or angiotensin receptor blocker (ARB) if ACEI is not tolerated) as the first-choice blood pressure lowering agent in people with diabetes and CKD stages 4 and 5 and micro/macroalbuminuria (Grade 1B). 6. We do not recommend the use of combinations of ACEIs and ARBs in people with diabetes and CKD stages 4 and 5 (Grade 2B). 7. We suggest dietary advice, correction of acidosis and loop diuretic therapy to lower serum potassium as necessary in people with diabetes and CKD stages 4 and 5 for safe use of ACEI (or ARB) (not graded). 8. Consider the use of novel potassium binders in people with diabetes and CKD stages 3b to 5 (non-dialysis) if potassium is 6 mmol/L or higher, for continued and safe use of ACEi (or ARB), or where people are not taking or are only taking sub maximal RAAS blockade because of hyperkalaemia (not graded). 9. We recommend dietary input to follow low sodium diet in all individuals with diabetes, advanced chronic kidney disease and high blood pressure (Grade 1B).	

#### Identification and monitoring of people with diabetes and CKD stages 4 and 5

The rise in blood pressure in people with diabetic nephropathy is associated with higher mortality and increased risk of macro and micro vascular complications [3, 186]; and treatment lowers cardiovascular events, strokes and all-cause mortality [187–189]. Hence people with diabetes and CKD stages 4–5 should be regularly screened to identify and manage high blood pressure. It is necessary to identify those with hypertension early to avoid delays in treatment; while avoiding unnecessary anxiety and the inconvenience related to frequent visits to doctors and nurses. With the use of RAAS blockers, monitoring of serum potassium is important to avoid dangerous hyperkalaemia [190]. Frequent blood testing will also identify people who are more likely to progress to renal replacement therapy [191]. Most clinical trials have monitored individuals’ clinical characteristics and laboratory values every 3 to 12 months and have demonstrated identification of new onset hypertension, proteinuria and hyperkalaemia in this time frame [184, 192, 193]. The recommendations for monitoring by NICE are: twice a year for CKD stage 3a, ≥2 times a year for CKD stage 3b, 3 times a year for CKD stages 3 and 4 and ≥ 4 times a year for CKD stage 5. However, this can be tailored according to the individual’s needs [138]. During the first consultation, ambulatory (or home) blood pressure monitoring should be offered to confirm the diagnosis of hypertension [34]. Measurement of sitting and standing blood pressure may be useful to diagnose postural hypotension which may contribute to symptoms and standing blood pressure may be a better target. Blood pressure should be measured by standardised technique in a quiet, comfortable environment, on an outstretched supported arm, using a properly calibrated machine with an appropriate cuff as suggested by the BIHS.

#### Target blood pressure in people with diabetes and CKD stages 4 and 5, with or without significant albuminuria [UACR > 3 mg/mmol (> 26.55 mg/g)]

Several observational and prospective studies have demonstrated the significant impact of blood pressure on mortality, cardiovascular events and renal failure in people with diabetes and CKD [3]. Among those from advanced CKD clinics, high blood pressure (particularly systolic) is associated with progression to dialysis and mortality [194].

Very few studies have examined the impact of tight blood pressure control in people with diabetes and CKD stages 4 and 5. Some studies have examined the role of intensive blood pressure lowering in people with diabetes and mild CKD; a small proportion of people demonstrating the advantage of lowering blood pressure below 140/90 mmHg but not below 130/80 mmHg [195]. In a study of African-American people with non-diabetes CKD and eGFR 20–65 mL/min/1.73 m^2^ the tight blood pressure [achieved 128/78 mmHg] control arm suffered similar renal end points compared to less tight blood pressure [achieved 141/85 mmHg] control arm [196]. In the SPRINT trial, which included 2646 non-diabetic participants with eGFR 20–60 mL/min/1.73 m^2^, intensive blood pressure control [target < 120 mmHg] was not associated with improved composite renal outcomes, compared with standard control in those with chronic kidney disease [target < 140 mmHg] [193]. However, no participants with diabetes were included in the SPRINT trial. In another large randomised controlled trial of high risk individuals with diabetes (ACCORD-BP), the intensive blood pressure control arm [target SBP < 120 mmHg; achieved 119 mmHg] was associated with a higher chance of having a eGFR < 30 mL/min/1.73 m^2^ [99 vs 52 events; *p* < 0.001] than the normal blood pressure control arm [target SBP < 140 mmHg; achieved 133 mmHg], without any benefit in reducing cardiovascular complications [192]. This trial excluded people with creatinine above 1.5 mg/dL (approximate eGFR 50 mL/min/1.73 m^2^), and mean creatinine at baseline was 0.9 mg/dL (approximate eGFR 91 mL/min/1.73 m^2^). In the same study there was no difference in new onset microvascular complications with intensive blood pressure control and half of the people who had progressive renal disease did not have albuminuria [197]. However, a pooled analysis of SPRINT and ACCORD-BP with 14,094 people followed for 3.26 years suggests a 18% risk reduction of cardiovascular events and cardiovascular deaths [198]. However, lowering blood pressure < 130/80 mmHg may be associated with unwanted side effects and individuals should be involved in the decision-making process [199].

Several studies have shown that presence of significant albuminuria is associated with poor cardiovascular outcomes and reduction of albuminuria is associated improvement [200]. Analysis of data from the RENAAL study, a trial of ARB in diabetic nephropathy, demonstrated an approximate doubling of risk of cardiovascular outcome with high albuminuria at baseline (UACR > 3 g/g compared with < 1.5 g/g of creatinine), and 18% lowering of the cardiovascular events with 50% lowering of albuminuria [201]. The evidence for better outcome with tighter blood pressure control with high albuminuria is mainly observational and derived from post hoc analysis of large randomised controlled trials. The RENAAL study which included a significant number of people with CKD stages 3 and 4 demonstrated baseline higher risk with SBP > 140 mmHg (no difference between < 130 compared with 130–140 mmHg), and a 23% risk reduction for ESKD with achieved BP < 140/90 compared with > 140/90 [202]. Analysis of data from two large ARB trials (IDNT and RENAAL) indicate that the benefits of cardiovascular risk reduction exist with SBP < 130 mmHg (particularly when albuminuria was reduced to lower levels). However, the risk increased with SBP < 120 mmHg [203]. Post-hoc analysis of IDNT trial demonstrated a benefit in reduction of heart failure events with SBP < 130 mmHg but possible increased risk with SBP < 120 mmHg; and a DBP < 85 mmHg was associated with increased risk of MI and CHF [204]. Hence there is a suggestion of better cardiovascular outcomes with reduction of systolic blood pressure below 130 mmHg but not below 120 mmHg. With target blood pressure < 130/80 mmHg in the STENO 2 randomised trial there was reduction in cardiovascular mortality; however most participants had CKD stages 1 and 2 and in the presence of other interventions it is difficult to tease out the effect of tight BP control [205]. Thus a lower target for blood pressure < 130/80 mmHg may be suggested in people with significant albuminuria as suggested by other guidelines (KDIGO/NICE) but stronger evidence is needed [138, 206]. The draft KDIGO 2020 guidelines suggest a lower blood pressure target < 120 mmHg systolic in all individuals with CKD with diabetes irrespective of the degree of proteinuria, while acknowledging that the degree of evidence in support of tighter control is low (evidence grade 2B), the clinical risk of adverse events with low blood pressure target, particularly when measured in non-standardised manner is high; thus allowing clinicians to target higher blood pressure when necessary [23]. This guideline proposes a target blood pressure of < 140/90 mmHg for all individuals with diabetes, no significant proteinuria and CKD stages 4 and 5, as improved cardiovascular outcomes have been demonstrated in randomised controlled trials with blood pressures < 140/90, but inconsistent results with lower targets. Whereas the proposed target is < 130/80 mmHg is for people with significant proteinuria as it is associated with reduction in proteinuria in diabetic kidney disease which may improve renal and cardiovascular outcomes.

#### Renin-angiotensin system blockade for blood pressure control in people with diabetes and CKD stages 4 and 5

In a study of African-American people without diabetes and with eGFR 20–65 mL/min/1.73 m^2^, use of the ACEI ramipril was associated with significant reduction in clinical composite outcome compared to metoprolol [22% (95% CI, 1–38%; *p* = 0.04)] or amlodipine [38% (95% CI, 14–56%; *p* = 0.004)] [196]. In a randomised controlled trial of people with diabetes [30% with mild CKD] use of enalapril was associated with fewer cardiovascular events compared with nisoldipine [5 vs. 25; *p* < 0.001] [207]. In a recent meta-analysis of 119 trials, use of ACEI or ARBs in 64,768 participants with CKD was associated with reduced risk of kidney failure compared with other antihypertensives [odds ratios of 0.65 (95% CI 0.51–0.80) for ACEIs and 0.75 (95% CI, 0.54–0.97) for ARBs] [208]. Hence ACEIs should be used in people with diabetes and CKD stages 4 and 5, with careful monitoring of kidney function and serum potassium. In a meta-analysis treatment with ACEIs in people with diabetes was shown to reduce reduce all-cause mortality, cardiovascular mortality and cardiovascular events but not with ARBs [209, 210]. A recent network meta-analysis showed reduction in ESKD with ACEI and/or ARB but did not demonstrate overall survival benefit [211]. Thus there is strong evidence for use of ACEI/ARB as the first choice antihypertensive in people with diabetes and CKD with eGFR > 30 mL/min/1.73 m^2^. Participants in large randomised controlled trials of people with diabetes had eGFR no lower than approximately 25 mL/min/1.73 m^2^ in REENAL and 38 mL/min/1.73 m^2^ in micro HOPE [202, 212]. Though hyperkalaemia and rapids decline in kidney function is an issue in people with advanced CKD, recent analysis of 3909 individuals with CKD stages 4–5 suggested continuing treatment with ACEI or ARB was associated with cardiovascular benefit. The impact of withdrawal of ACEI or ARB on progression of CKD is being investigated by the STOP ACE randomised controlled trial [213, 214].

In a study of combination therapy of ACEI with ARB in people who have diabetes with UACR > 33.9 mg/mmol (300 mg/g) and eGFR 30–90 mL/min/1.73 m^2^, there was no difference in mortality but a significant in increase in hyperkalaemia [6.3 events vs. 2.6 events per 100 person-years with monotherapy; *p* < 0.001] and acute kidney injury [6.7 vs. 0.2 events per 100 person-years, *p* < 0.001] [80]. Combination of ACEI with ARB was not associated with benefit in primary endpoints but more side effects hence should be avoided. However, a network meta-analysis suggested a potential benefit of dual blockade if it can be administered safely, hence the need for further trials of dual-blockade in diabetes patients with CKD and albuminuria [146, 211].

A rise in serum creatinine up to 30% is not uncommon and rise of potassium by 0.5 mmol/L is not uncommon with initiation of ACEI therapy [85]. A post-hoc analysis of the ACCORD-BP trial demonstrates > 30% rise in creatinine identifying patients at risk for cardiovascular and all-cause mortality, but only associated with adverse renal outcome in the standard arm and not intensive therapy arm [198]. Thus a mild rise in creatinine may not require any change in planned therapy. Hence no modification of ACEI or ARB therapy is necessary if the rise in creatinine from baseline is < 30% or drop in eGFR is < 25%.

Addition of spironolactone and further inhibition of the renin-angiotensin-aldosterone system may provide additional anti-proteinuric effect as seen in small studies and merits further large trials with more clinically relevant outcomes [215].

### Management of hyperkalaemia with renin-angiotensin system blockade in people with diabetes and CKD stages 4 and 5

Hyperkalaemia is common in people with diabetes and CKD. It is very common (> 30%) in advanced CKD patients managed in the low-kidney-clearance clinics [182]. The cause of such hyperkalaemia can be multifactorial; including renal failure, type 4 renal tubular acidosis, diet and drugs. The presence of hyperkalaemia limits the use of renin-angiotensin-axis inhibitors. The chronically high potassium levels have been traditionally controlled with restricted diet, diuretics and avoiding drugs that cause hyperkalaemia.

Traditionally hyperkalaemia has been managed with dietary potassium restriction and correction of acidosis, if present with bicarbonate therapy. However, the new potassium binding agents have been tested for safety and efficacy in randomised controlled trials for management of chronic hyperkalaemia in CKD patients. They cause an early and sustained lowering of potassium in people with CKD on RAAS blocker therapy [216]. In 306 individuals with diabetes and CKD stages 3 to 4, treated with RAAS blockade [ACEI/ARB ± spironolactone], use of a novel potassium binding polymer (patiromer) was associated with significant and sustained decrease in serum potassium over 52 weeks [217]. In a study of 237 participants with CKD the same potassium binder was able to reduce serum potassium by 1 mmol/L over 4 weeks [218]. In another study of 243 participants over > 50% of whom had diabetes, the potassium binder achieved approximately 1 mmol/L reduction in serum potassium over 4 weeks in individuals with and without heart failure [219]. The treatment with patiromer was associated with decrease aldosterone levels and decreased blood pressure which may provide additional benefits [220]. However, the above-mentioned trials are of short duration and the possible ACEI or ARB use facilitated with potassium binders, has not been shown to improve cardiovascular events or mortality. However, in the recent AMBER study, in people with resistant hypertension and chronic kidney disease (50% with diabetes), patiromer enabled more individuals to continue treatment with spironolactone with less hyperkalaemia [221, 222].

Novel potassium binders may be useful in diabetes patients with chronic kidney disease, particularly when associated with left ventricular dysfunction. Recent NICE technology appraisals recommend the use of potassium binders, patiromer and sodium zirconium cyclosilicate, in outpatient care for people with persistent hyperkalaemia (> 6 mmol/L) and CKD stages 4 to 5 (non-dialysis), who are not taking an optimised dosage of RAAS inhibitor because of hyperkalaemia [162, 163].

#### Non-pharmacological management of hypertension in people with diabetes and CKD stages 4 and 5

In randomised controlled trials dietary sodium restriction in people with CKD is associated with significant lowering of blood pressure, but longer-term benefits of dietary intervention are unknown [223, 224]. The dietary advice is best provided by a trained dietitian due to the complex and frequently changing needs in this group of people. Individuals with CKD stages 4 and 5 would benefit most from this and are best managed in a multidisciplinary clinic with expert nurses and dietitians. Dietary potassium restriction is useful but clinical trial evidence is yet to be generated. Regular exercise tends to improve quality of life, eGFR decline, HbA1c, BMI without any adverse effects in people with diabetes and CKD stages 3–5 [225]. People should be also advised to quit smoking which is known to improve blood pressure in hypertensive individuals. Please also see Table 4.

### Hypertension management and renin-angiotensin-aldosterone system blockade in people with diabetes and CKD stage 5 on dialysis (5D)

#### Recommendations

Hypertension is a common finding in people with diabetes as well as those with CKD stage 5D. Elevated blood pressure [226, 227], diabetes [3, 228] and CKD [229–231] are all major risk factors for adverse cardiovascular events.

According to the UK Renal Registry and the European Renal Association – European Dialysis and Transplant Association (ERA-EDTA) Renal Registry, 23–36% of incident dialysis patients had diabetes as their primary renal disease [232, 233]. People with diabetes who are on haemodialysis have a poorer survival rate compared with dialysis patients who do not have diabetes [234, 235]. This is mainly due to cardiovascular disease [236–238]. Control of hypertension in hypertensive dialysis patients was shown to be associated with improved survival [239].

It is therefore logical that, in order to reduce cardiovascular risk and improve survival, optimal blood pressure control should be achieved in people with diabetes and CKD stage 5D. However, there is insufficient evidence from data in the published literature to decide how best to manage blood pressure in people with diabetes who are on dialysis. This is in part because people with CKD, including those with stage 5D, are ‘often’ excluded from clinical trials of hypertension.

There are emerging, although not consistent, data delineating how best to measure blood pressure, to target blood pressure and to use pharmacological and non-pharmacological therapies to optimise blood pressure control in people with CKD stage 5D. However, these data are not specific to the population with diabetes.

Furthermore, blood pressure control in people who are on dialysis is complex. Many factors affect blood pressure in people who are on dialysis, including fluid status, salt intake, sympathetic nervous system activity and the renin-angiotensin-aldosterone system (RAAS). People with diabetes who are undergoing haemodialysis often have autonomic dysfunction [240], which increases the risk of cardiovascular instabilities during dialysis. Haemodialysis causes severe orthostatic reduction in cerebral blood flow velocity in people with diabetes and may subsequently increase the risk of cerebrovascular injury post haemodialysis [241]. This makes management of hypertension in people with diabetes who are on dialysis even more challenging (Table 6).

**Table 6:** Recommendations for people with type 2 diabetes on dialysis

Recommendations for hypertension management and RAAS blockade in people with diabetes on modialysis	
1. We recommend that ambulatory blood pressure measurement or home blood pressure measurement should be used to monitor blood pressure in people with diabetes who are on dialysis (Grade 1C). 2. Where ambulatory blood pressure measurement or home measurement are not feasible to monitor blood pressure in people with diabetes who are on dialysis, we suggest using pre-, intra- and post-dialysis standardised blood pressure measurements for people who are on haemodialysis, and using standardised clinic blood pressure measurements for people who are on peritoneal dialysis (Grade 2D). 3. We recommend volume control as a first-line management to optimise blood pressure control in people with diabetes who are on dialysis (Grade 1B). 4. We suggest salt restriction to < 5 g per day to optimise blood pressure control in people with diabetes who are on dialysis (Grade 2C). 5. We suggest a target upright interdialytic blood pressure of < 140/90 mmHg for people with diabetes who are on dialysis. Individualisation of the blood pressure target may be indicated in other people who are burdened with multiple comorbidities, in order to reduce adverse events of blood pressure lowering (Grade 2D). For peritoneal dialysis patients we also suggest the target BP is < 140/90 mmHg (Grade 2D) 6. We recommend that intradialytic hypotension should be avoided in people with diabetes who are on haemodialysis (Grade 1B). 7. We suggest using ACEIs or ARBs (but not in combination), beta blockers and calcium channel blockers to reduce cardiovascular complications in people with diabetes and hypertension who are on dialysis (Grade 2B). 8. We suggest the use of diuretics in people with diabetes who are on dialysis and have residual renal function (Grade 2C).	

#### Blood pressure measurement in people with diabetes who are on haemodialysis

In UK dialysis units, measuring pre- and post-dialysis blood pressure is the standard technique for monitoring blood pressure in people who are on dialysis. However, blood pressure measurement in people who are on haemodialysis is complex. There are conflicting data as to whether blood pressure measurements pre- and post-dialysis are predictive of interdialytic blood pressure in comparison with ambulatory blood pressure measurement and/or home blood pressure measurement. Ambulatory blood pressure measurement is considered to be the most accurate method for studying blood pressure in people who are on haemodialysis [242] and in the general population it provides a more accurate prediction of cardiovascular outcomes in comparison with clinic blood pressure measurement [243]. A meta-analysis of 18 studies that involved 692 people who were on dialysis showed that pre-dialysis blood pressure and post-dialysis blood pressure are imprecise estimates of interdialytic ambulatory blood pressure [244]. People with diabetes were included in most of these studies [236, 242] at a rate that varied from 8 to 54%. The presence of diabetes made no difference to the outcome.

In an extensive review of the literature by Agarwal et al [245], evidence from several studies was presented to show that, in people on haemodialysis, blood pressure measurement at home [246] or ambulatory blood pressure measurement [247, 248] are stronger predictors of LVH [249] and mortality [250, 251] compared with blood pressure obtained in the dialysis unit. In predicting LVH, weekly average home systolic blood pressure measurement was similar to interdialytic ambulatory blood pressure measurement and was superior to pre-dialysis and post-dialysis blood pressure measurement [249]. In contrast to home blood pressure measurement, ambulatory blood pressure measurement can diagnose nocturnal non-dipping and offers great insights into circadian rhythm [252]. Loss of diurnal rhythm, which is a feature of diabetic nephropathy, is reported to lead to worse outcomes in people who are on dialysis [247]. In a study of 89 people who are on haemodialysis by Liu et al, the incidence of cardiovascular events and deaths were 3.5–9 times higher in non-dippers (that is, those who lose their diurnal blood pressure variation) compared with dippers [253]. Ambulatory blood pressure measurement can therefore be advantageous in selecting high-risk individuals and can guide treatment. However, to date, there have been no specific studies to address ambulatory blood pressure measurement in people with diabetes who are on dialysis.

While 24-h ambulatory blood pressure measurement (ABPM) is considered gold standard in predicting outcomes, it is resource intensive, impractical in for long-term monitoring of BP control and often not tolerated by individuals. Interdialytic home blood pressure (HBPM) is close to ABPM in predicting outcomes, but has high attrition rate for long-term monitoring. As routinely collected peri-dialytic BP measurements guide interventions in the majority of dialysis units, it is essential to ensure routinely collected dialysis unit BP readings are measured in a standardised manner in accordance with recommended guidelines.

#### Target blood pressure in people with diabetes who are on dialysis

The relationship between blood pressure level and cardiovascular outcome is unclear in individuals who are on dialysis. Observational studies have shown an increased risk of mortality in people who are on haemodialysis who have a low pre- or post-dialysis systolic blood pressure of < 110 mmHg [254], and in those who are on peritoneal dialysis with a pre-dialysis systolic blood pressure of < 110 mmHg [255]. Further observational studies in haemodialysis cohorts [256, 257] continued to show a reverse epidemiology phenomenon, with the highest mortality rate being in groups with lower pre-dialysis blood pressures. Recent observational relationship studies using peri-dialytic measurements have consistently shown a ‘U’- or ‘J’-shaped relationship with mortality. For example, analysis of Dialysis Outcomes and Practice Patterns Study (DOPPS) data, at both facility and individual patient level, found lowest mortality in those with a pre-HD SBP of 130 to 159 mmHg (facility level) and < 130 mmHg (individual patient level); the facility-level analysis compensates for unmeasured confounding, albeit in an imperfect manner [258]. Similarly, the CRIC Investigators reported a pre-HD SBP of 138 to 166 mmHg to be associated with lowest risk of cardiovascular events [259].

Interestingly, in the Tassin group in France where the 5-year survival rate of 87% is the best reported in people who are on haemodialysis, the pre-dialysis blood pressure that was achieved was < 130/85 mmHg (mean arterial pressure (MAP) < 101 mmHg) [260].

Prospective randomised controlled studies on the effect of ARBs [261], ACEIs [262], beta blockers [263] and calcium channel blockers [264] on cardiovascular events have been conducted to evaluate the roles of these agents in people who are on dialysis.

Two meta-analyses have shown that blood pressure treatment in people who are on dialysis is associated with improved outcome. The first analysis was by Heerspink et al, published in 2009 [265]. This meta-analysis included eight randomised trials that provided data from 1679 people who are on dialysis, of whom 588 had diabetes. The trials included people who were on haemodialysis and peritoneal dialysis. The analysis showed that blood pressure lowering treatment was associated with lower risks of cardiovascular events, all causes of mortality and cardiovascular mortality, and that the effect seemed to be consistent across a range of groups that were included in the studies. Reduction in systolic blood pressure was similar, regardless of whether the person had diabetes or antihypertensive drug use. Similarly, the second meta-analysis by Agarwal and Sinha (also published in 2009) [266] showed that in people with hypertension who were on haemodialysis, antihypertensive therapy reduced the combined hazard ratio for cardiovascular events by 31–38% compared with the placebo group. The meta-analysis showed that blood pressure lowering was well tolerated, with no suggestion of increased adverse events in people with diabetes. The analysis showed no difference in cardiovascular outcomes caused by different drug classes and the data from the two meta-analyses suggest that RAAS blockers, beta blockers and calcium channel blockers are all suitable for use in people who are on dialysis.

However, a randomised controlled trial by Agarwal et al in 2014 [267], including 200 people who were on haemodialysis of whom nearly half had diabetes, showed that a beta blocker-based hypertensive treatment was superior to an ACEI-based treatment in preventing cardiovascular morbidity in those who are on dialysis.

Irrespective of the type of the antihypertensive agents that are used, the timing of the administration of such agents in relation to dialysis treatment needs to be taken into account when prescribing antihypertensive drugs for people who are on dialysis. ARBs, calcium channel blockers and alpha-blockers are not cleared with dialysis. However, ACEIs (apart from fosinopril) and a number of beta blockers are largely cleared on dialysis [268].

To date, optimum blood pressure goals for individuals who are on dialysis (including people with diabetes) have not been defined in randomised prospective controlled trials [268]. The Kidney Disease Outcomes Quality Initiative (KDOQI) recommends a pre-dialysis blood pressure goal of < 140/90 mmHg and a post-dialysis blood pressure goal of < 130/80 mmHg [269]. However, this is largely based on studies that were performed in the non-dialysis population who have normal renal function. People who are on haemodialysis have different characteristics to the general population. For example, studies have shown that people who are on dialysis lose their diurnal blood pressure variation (that is, they are non-dippers), which is an independent risk factor for LVH and subsequent adverse cardiovascular outcome [247]. People who are on haemodialysis also have increased pulse pressure, which is associated with adverse cardiovascular outcome [270]. Therefore, the KDOQI-recommended blood pressure target may not be applicable to the haemodialysis population.

Furthermore, people with diabetes who are on dialysis are at increased risk of haemodynamic instabilities and orthostatic intolerance post-dialysis, and therefore a blood pressure that is higher than 140 mmHg systolic may be indicated in the presence of significant orthostatic change in blood pressure. A randomised controlled trial is needed to identify the optimal blood pressure target for people with diabetes who are on dialysis.

As for those who are on peritoneal dialysis, International Society of Peritoneal Dialysis (ISPD) guidelines recommend a target of < 140/90 mmHg for self-measured home blood pressure readings [271]. However, this is based on a number of small observational studies and not specifically for those who have diabetes. There is no randomised controlled trial evidence currently available.

#### Volume control in people with diabetes who are on dialysis

Increased extracellular volume or volume overload is an important contributor in the pathogenesis of high blood pressure in people who are on dialysis [272]. Removal of extracellular volume without causing intolerable hypotension defines the ‘dry weight’ [273] that was first reported by Thomson in 1967 [274]. This is difficult to define clinically. Achieving dry weight and normalising blood pressure is not immediate and can take months, which is something that is best described as a ‘lag phenomenon’ [275].

In the Tassin group, hypertension control without medication, achieved by aggressive control of extracellular volume and dietary sodium intake, was shown to be the best single marker of survival in 449 people who were on haemodialysis who were followed for 20 years [260].

Observational studies showed that volume control is associated with improvement in blood pressure in the majority of people on haemodialysis [276] and peritoneal dialysis [277]. A randomised controlled trial (DRIP) showed that volume control in haemodialysis improves blood pressure control [278]. In that study, 150 people were randomised to an additional ultrafiltration group (40/100 had diabetes) or control group (19/50 had diabetes). Without increasing time or frequency of haemodialysis, reduction in dry weight (defined by clinical signs and symptoms) resulted in a reduction in interdialytic ambulatory blood pressure, leading to the conclusion that dry weight reduction is an effective strategy in blood pressure control in people who are on haemodialysis.

The concept that ‘volume control’ improves blood pressure control is further supported by the increasing reports that daily dialysis [279, 280] or nocturnal dialysis [281, 282] improves blood pressure and reduces LVH with less risk of inducing intradialytic hypotension. Reducing the risk of intradialytic hypotension is important. An observational study by Shoji T et al showed that haemodialysis-associated hypotension is an independent risk factor for 2-year mortality in people who are on haemodialysis [283].

The risk of intradialytic hypotension increases with an ultrafiltration rate of > 10 ml/kg/hr. and was reported in the Dialysis Outcomes and Practice Patterns Study (DOPPS) (which included 16,420 patients on haemodialysis) as an independent risk factor for mortality [284]. This is similar to another study with 5 years’ follow-up data by Movilli [285], in which an ultrafiltration rate of over 12.7 ml/kg/hr. was identified as an independent risk factor for mortality given the risk of hypotension-related serious adverse events especially in people who are on dialysis and who have diabetes; however, this strategy requires close supervision and markers to assess volume status.

Bioimpedance spectroscopy devices [286], brain natriuretic peptide (BNP) level [287], assessment of vena cava diameter [288] and ultrasound lung water measurement [289] have been used to determine dry weight. Of these, bioimpedance spectroscopy is most widely studied [290]. Further studies are needed to explore and evaluate the role of bioimpedance spectroscopy devices as markers of volume status, especially in people with diabetes who are on dialysis.

#### Salt restriction in people with diabetes who are on dialysis

Reducing dietary salt to control blood pressure in people who are on dialysis was first reported by Hegstrom RM et al in 1961 [291]. Salt restriction to 1 g per day or less helps to decrease thirst and to control interdialytic weight gain in people who are on haemodialysis [292].

Evidence for the association between salt restriction and blood pressure control in people who are on dialysis comes from observational studies where dietary salt restriction was in combination with strict volume control. Craswell et al [293] (in a study of 89 people who were on dialysis), Covic et al [294] (in a study of 286 people) and Ozkahya et al [295] (in a study of 218 people) all showed that salt restriction to < 5 g per day along with strict volume control led to a significant reduction of blood pressure and interdialytic weight gain. Similarly, in the Tassin group, dietary salt reduction to < 5 g per day along with extracellular volume control was shown to normalise blood pressure in people who were on haemodialysis [260].

In a cross-sectional study by Kayikcioglu et al in 204 people on dialysis, dietary salt restriction to 5 g per day along with dialysate sodium reduction, led to a reduction in interdialytic weight gain, the number of antihypertensive medications and LVH [296]. Maduell and Navarro (in a cross-sectional study of 15 people) reported that salt restriction alone resulted in a significant reduction in interdialytic weight gain and blood pressure [297]. In the Haemodialysis (HEMO) Study, dietary sodium intake was associated with a greater adjusted risk of all-cause mortality [298]. In practical terms, adherence may be more sustainable if a threshold restriction of < 6 g dietary salt is applied in the diabetes cohort who have additional restrictions placed on them, but this has yet to be formally evaluated.

Diuretic therapy may provide an additional means by which to promote natriuresis in people who are on dialysis who have residual urine output. In the DOPPS study, Bragg-Gresham et al reported that diuretic use was associated with reduced interdialytic weight gain, fewer intradialytic hypotensive episodes and reduced cardiac-specific mortality, but not all-cause mortality [284]. People with residual renal function who were on diuretics were twice as likely to retain residual renal function compared with those who were not on diuretics after 1 year in the study. The authors concluded that people with residual renal function may benefit from continuing diuretic use rather than automatically discontinuing it at the start of dialysis. Furthermore, in a prospective randomised study by Medcalf et al on people who are on peritoneal dialysis, frusemide given at a dose of 250 mg once daily produced clinically significant preservation in urine volume over 1 year, but it had no influence on residual renal function [299].

Interestingly, and independent of its diuretic property, spironolactone has been shown, in a randomised controlled trial that included participants with diabetes, to be more effective than placebo in treating refractory hypertension in people on dialysis [300]. There is emerging evidence from a number of randomised controlled trials that spironolactone has a cardiac protective effect in people on dialysis [301], but it will be interesting to see what emanates from the current ongoing larger randomised controlled trial (ALdosterone Antagonist Chronic HEModialysis Interventional Survival Trial (ALCHEMIST)), which is exploring the potential cardiac protective role of spironolactone in people who are on dialysis [302]. It might help to show whether this effect is dependent or independent of spironolactone’s property as a diuretic and/or antihypertensive agent.

To date there have been no randomised controlled trials to address the question of whether salt restriction or diuretic use in people with diabetes who are on dialysis may influence blood pressure control or cardiovascular outcome. In the absence of such evidence, individualisation of dietary sodium intake is required, depending on the person’s interdialytic weight gain, extracellular volume status, haemodynamic stability and nutritional status.

### Research recommendations

There are areas which require further investigation including randomized controlled trials to further improve care of people with diabetes and CKD as highlighted in Table 7.

## Conclusion

People with diabetes and CKD have increased risk of morbidity and mortality. They experience excess cardiovascular events and progression of CKD. Hypertension is a common risk factor for both adverse outcomes. Tight control of blood pressure and the use RAAS inhibitors are associated with improved outcomes, particularly in the presence of proteinuria. RAAS inhibitors can cause side effects e.g. rising serum creatinine and hyperkalaemia. However, guideline-based therapy such as mentioned here can prolong life, lower risk of cardiovascular events and hospital admissions, and prevent end-stage kidney failure requiring renal replacement therapy.

### Research recommendations

**Table 7:** The main research recommendations

The future research recommendations for type 1 and type 2 diabetes with different stages of CKD	
The following areas lack good-quality evidence for RAAS blockade and hypertension management in people with type 1 diabetes, and hence further research is necessary. 1. In light of the fact that the presence of microalbuminuria in people with type 1 diabetes may not be the best predictor of whether they will develop progressive renal disease, what is the role for other markers (such as kidney injury molecule-1 (KIM-1)) in predicting the risk of renal disease in those with type 1 diabetes? 2. What is the role of dual RAAS blockade in people with type 1 diabetes and nephropathy? 3. What is the role of aldosterone receptor blockers in people with type 1 diabetes and nephropathy? 4. Is there a role for home or ambulatory blood pressure monitoring in the diagnosis and management of hypertension in people with type 1 diabetes, particularly in those who have diabetic autonomic neuropathy? 5. Does measurement of plasma renin activity have a role in screening and managing hypertension in people with type 1 diabetes? 6. Does tight glycaemic control and blood pressure lowering reduce the incidence of people developing microvascular complications in type 1 diabetes? 7. What is the role of RAAS-blocking agents in people who have type 1 diabetes, progressive renal decline and normoalbuminuria? 8. What is the impact on renal function of lower blood pressure targets in younger people with type 1 diabetes and nephropathy?	
The following areas lack good-quality evidence for RAAS blockade and hypertension management in people with type 2 diabetes, nephropathy and/or early CKD, and hence further research is necessary. 1. What is the best method for blood pressure measurement in people with type 2 diabetes who have CKD, particularly those with autonomic neuropathy? 2. What is the evidence-based lower limit for blood pressure reduction (< 130/80 mmHg) in people with type 2 diabetes who have CKD in terms of cardiovascular and renal endpoints? 3. Can novel potassium binders enable a higher dosage of RAAS inhibitors or dual blockade with better attainment of blood pressure control and improvement in cardiovascular and renal outcomes? 4. What are the best second- and third-line blood pressure lowering agents in people with type 2 diabetes who have CKD and proteinuria? 5. Is there a need for long-term outcome studies of non-dihydropyridine calcium channel blockers in diabetic nephropathy? 6. Does bedtime hypertension treatment improve cardiovascular and renal outcomes in patients with type 2 diabetes and CKD? 7. What is the role of lifestyle modifications (such as salt restriction, regular exercise, weight reduction) on blood pressure control, and cardiovascular and renal outcomes?	
The following areas lack good quality evidence and further research may help in people with diabetes on dialysis 1. Which blood pressure measurement should be used to predict left ventricular hypertrophy (LVH) and mortality in people with diabetes who are on dialysis: pre-dialysis, post-dialysis, home or ambulatory blood pressure measurement? 2. What is the optimal upright blood pressure target for people with diabetes who are on dialysis? 3. Can bioimpedance spectroscopy devices be used to determine a target weight and predict the risk of cardiovascular morbidity for people with diabetes who are on dialysis? 4. Does treatment with ACEIs, ARBs, beta blockers or calcium channel blockers to lower blood pressure in people with diabetes who are on dialysis reduce cardiovascular morbidity and mortality? 5. Is there a role for diuretic therapy in people with diabetes who are on dialysis and have residual renal function? 6. Does salt restriction (< 5 g per day) in people with diabetes who are on dialysis influence blood pressure control or cardiovascular outcome?	

## Supplementary Information

**Additional file 1.**

## Data Availability

Not applicable.

## Abbreviations

ABCD: Association of British Clinical Diabetologists
ACE: Angiotensin converting enzyme
ABPM: Ambulatory blood pressure monitoring
AER: Albumin excretion ratio
ARB: Angiotensin receptor blockers
BMI: Body mass index
BP: Blood pressure
CKD: Chronic kidney disease
eGFR: Estimated glomerular filtration rate
HBPM: Home blood pressure monitoring
KDIGO: Kidney Disease Improving Global Outcome
LVH: Left ventricular hypertrophy
MAP: Mean arterial pressure
NICE: National Institute for Health and Care Excellence
RAAS: Renin angiotensin aldosterone system
SBP: Systolic blood pressure
SGLT-2: Sodium glucose transporter 2
UACR: Urine albumin creatinine ratio
UK: United Kingdom
