# Supplementary material for: Management of hypertension and renin-angiotensin-aldosterone system blockade in adults with diabetic kidney disease: Association of British Clinical Diabetologists and the Renal Association UK guideline update 2021
Source: BMC Nephrol. 2022 Jan 3;23:9. doi: 10.1186/s12882-021-02587-5 (PMC8722287; doi:10.1186/s12882-021-02587-5)
Supplement: Supplementary file 1 [file 12882_2021_2587_MOESM1_ESM.odt]

# Supplementary Files:

### Search strategy

The recommendations are based on a review of literature carried out initially between October 2013 and December 2016 and a further review carried out till December 2020 for the current update. We searched the Cochrane Library, PubMed/MEDLINE, Google Scholar and Embase, using the following key terms: type 1 diabetes, type 2 diabetes, hypertension, blood pressure, albuminuria, microalbuminuria, proteinuria, microvascular complications, nephropathy, chronic kidney disease, therapy, angiotensin-converting enzyme inhibitors, angiotensin receptor blockers and mineralocorticoid antagonists.

### Supplementary table 1: The grades of evidence 

| The recommendations and evidence base |
| --- |
| 1A – Strong recommendation: high-quality evidence 1B – Strong recommendation: moderate-quality evidence 1C – Strong recommendation: low-quality evidence 1D – Strong recommendation: very low-quality evidence |
| 2A – Weak recommendation: high-quality evidence 2B – Weak recommendation: moderate-quality evidence 2C – Weak recommendation: low-quality evidence 2D – Weak recommendation: very low-quality evidence |

Legend: The recommendations, 1 or strong and 2 weak (suggestion only) with the grade of evidence, A to D depending on quality of evidence

### Supplementary table 2: Audit standards for RAAS blockade and hypertension in people with type 1 and type 2 diabetes and different stages of CKD  

| The following are suggested as audit standards for RAAS blockade and hypertension management in people with type 1 diabetes |
| --- |
| . The proportion of people with type 1 diabetes, with micro- or macroalbuminuria who are treated with an ACEI or ARB at the maximum tolerated doses. The proportion of people with type 1 diabetes with micro- or macroalbuminuria who are achieving a blood pressure of less than 130/80 mmHg for younger adults or less than 140/90 mmHg for those aged over 65 years. The proportion of younger adults (under 30 years old) with persistent albuminuria who are achieving systolic blood pressure of less than 120 mmHg The proportion of people with type 1 diabetes and albuminuria who are on submaximal doses of ACEIs or ARBs due to hyperkalaemia or adverse reactions. |
| The following are suggested as audit standards for RAAS blockade and hypertension management in people with type 2 diabetes, early CKD and/or proteinuria. |
| The percentage of people with type 2 diabetes who have CKD and urine AER of greater than 30 mg per 24 hours (ACR of greater than 3 mg/mmol) who are achieving the target upright blood pressure of less than 130/80 mmHg. The proportion of people with type 2 diabetes who have CKD and urine AER of greater than 30 mg per 24 hours (ACR of greater than 3 mg/mmol) who are on ACEIs or ARBs. The percentage of people with type 2 diabetes who have CKD and urine AER of greater than 30 mg per 24 hours (ACR of greater than 3 mg/mmol) who are not on ACEIs or ARBs (or who are on a submaximal dosage) due to hyperkalaemia or a decrease in estimated glomerular filtration rate (eGFR) of greater than 25% or an increase in serum creatinine of greater than 30%. The number of people with type 2 diabetes and CKD who are on dual blockade of the RAAS. |
| The following are suggested as audit standards for management of hypertension and RAAS blockade in people with diabetes and CKD stages 4 and 5 |
| Proportion of people with blood pressure <140/90 mmHg with diabetes and CKD stages 4 and 5 with ACR <3 mg/mmol. Proportion of people with blood pressure <130/80 mmHg with diabetes and CKD stages 4 and 5 with ACR >3 mg/mmol. Proportion of people who are taking an ACEI/ARB and have a serum potassium  >5.5 mmol/L. Proportion of people with potassium >5.5 mmol/L and being seen by dietitian. |
| The following are suggested as audit standards for management of hypertension and RAAS blockade in people with diabetes in dialysis |
| The proportion of people with diabetes who are on dialysis who achieve an upright interdialytic blood pressure target of less than 140/90 mmHg. The proportion of people with diabetes who are on dialysis who achieve an upright interdialytic blood pressure target of less than 140/90 mmHg without the use of blood pressure lowering medication. The proportion of people with diabetes who are on dialysis who have an interdialytic blood pressure of greater than 140/90 mmHg who are not being treated with ACEIs, ARBs, beta blockers or calcium channel blockers in whom such drugs are not contraindicated. The proportion of people with diabetes who are on dialysis and on diuretics following commencement of dialysis. |
